# Wavelet-based whole genome doubling aware single-cell copy number calling

**DOI:** 10.1093/nar/gkag960

**Published:** 2026-10-09

**Authors:** Benjamin K Wesley, Frank Wos, Soren Germer, Jade E B Carter, Silas Maniatis, Khanh Dinh, James S Roche, Timothy R Chu, Nicolas Robine, Rebecca C Fitzgerald, Lizhe Zhuang, Simon Tavaré, Karol Nowicki-Osuch

**Affiliations:** Irving Institute for Cancer Dynamics, Columbia University, Room 601 Schermerhorn Hall, 1190 Amsterdam Ave, New York, NY 10027, United States; Department of Biological Sciences, Columbia University, Room 600 Sherman Fairchild Center, 1212 Amsterdam Ave, New York, NY 10027, United States; New York Genome Center, 101 Avenue of the Americas, New York, NY 10013, United States; New York Genome Center, 101 Avenue of the Americas, New York, NY 10013, United States; New York Genome Center, 101 Avenue of the Americas, New York, NY 10013, United States; New York Genome Center, 101 Avenue of the Americas, New York, NY 10013, United States; Irving Institute for Cancer Dynamics, Columbia University, Room 601 Schermerhorn Hall, 1190 Amsterdam Ave, New York, NY 10027, United States; Department of Statistics, Columbia University, 1255 Amsterdam Ave, New York, NY 10027, United States; New York Genome Center, 101 Avenue of the Americas, New York, NY 10013, United States; New York Genome Center, 101 Avenue of the Americas, New York, NY 10013, United States; New York Genome Center, 101 Avenue of the Americas, New York, NY 10013, United States; Early Cancer Institute, University of Cambridge, Clifford Allbutt Building, Adrian Way, Cambridge Biomedical Campus, Cambridge CB2 0AH, United Kingdom; Early Cancer Institute, University of Cambridge, Clifford Allbutt Building, Adrian Way, Cambridge Biomedical Campus, Cambridge CB2 0AH, United Kingdom; Irving Institute for Cancer Dynamics, Columbia University, Room 601 Schermerhorn Hall, 1190 Amsterdam Ave, New York, NY 10027, United States; Department of Biological Sciences, Columbia University, Room 600 Sherman Fairchild Center, 1212 Amsterdam Ave, New York, NY 10027, United States; Department of Statistics, Columbia University, 1255 Amsterdam Ave, New York, NY 10027, United States; German Cancer Research Center (DKFZ) Heidelberg, Tumorigenesis and Molecular Cancer Prevention Group, Im Neuenheimer Feld 280, Heidelberg 69120, Germany; DKFZ Hector Cancer Institute at the University Medical Center Mannheim, Im Neuenheimer Feld 280, Heidelberg, 69120, Germany

## Abstract

Advances in single-cell whole genome sequencing enable profiling of the copy number state of thousands of cells with minimal sequencing bias across the genome. The Direct Library Preparation+ technique is a whole genome amplification-free single-cell whole genome sequencing method that achieves high throughput by fragmenting each cell’s genome and ligating sequencing adapters using a modified Tn5 transposase, and sequencing to <0.1× coverage. Despite recent advances in experimental approaches, data analysis of single-cell whole genome sequencing lags behind and the existing methods are not optimized for the analysis of frozen samples with variable DNA preservation. Furthermore, existing tools predominantly rely on read depth ratio in predefined genomic bins to call copy number, making whole genome duplication unidentifiable. To address this, we introduce Songbird, a single-cell whole genome sequencing copy number caller that is whole genome duplication sensitive, and outperforms existing tools both in breakpoint identification and true copy number detection. We demonstrate that Songbird is robust down to very low coverage, adaptable to a variety of genome versions (hg19, hg38, hs.1), and is extensible to other single-cell whole genome sequencing methods that rely on Tn5 tagmentation to fragment the genome.

## Graphical Abstract

**Graphical Abstract:**
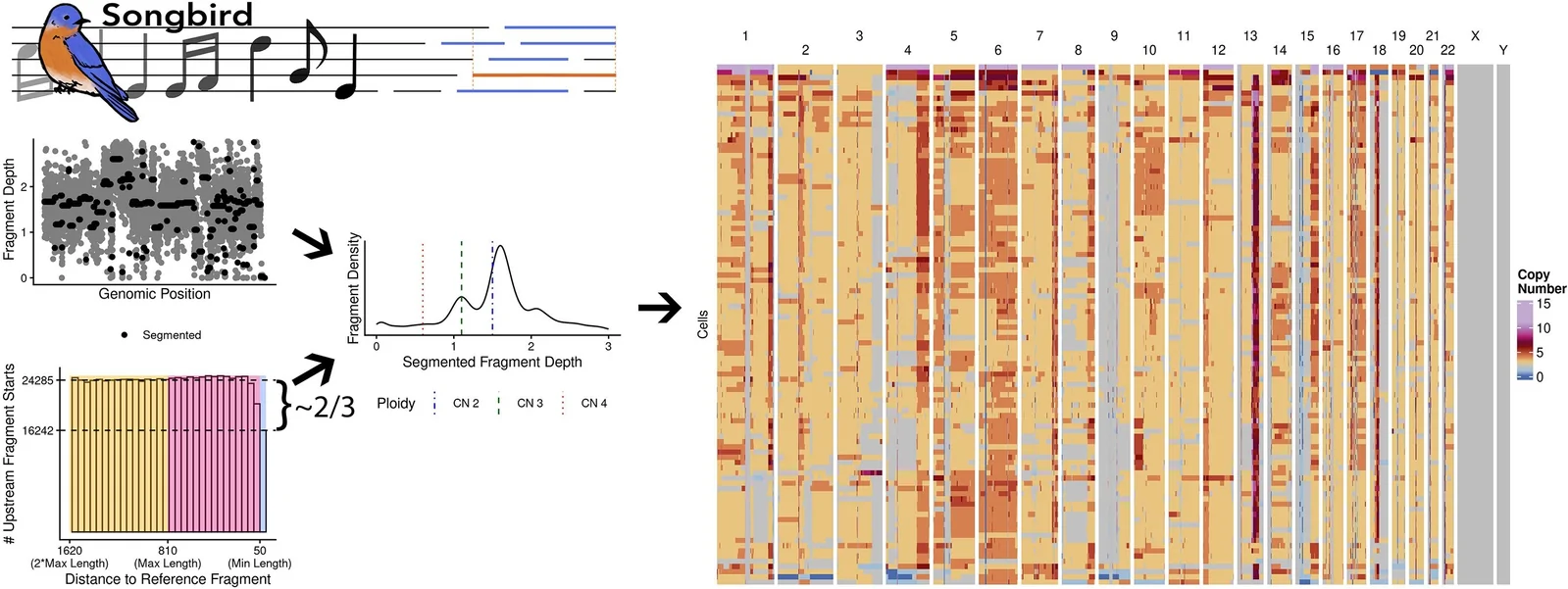

## Introduction

Copy number variations (CNVs) are genome-wide changes observed across the majority of solid tumor types and often contribute significantly to the fitness advantage exhibited by primary tumor subclones [1]. CNVs can arise in many ways, but oncogene-driven whole genome doubling (WGD) is thought to be a common pathway to aneuploidy as it enables cells to survive chromosomal rearrangements without suffering haploinsufficiency of essential genes [2]. For example, gastric cancers and esophageal adenocarcinomas often undergo WGD early in their progression [3]. Alternatively, failures in DNA damage response enzymes or micronuclei formation cause rearrangements and genomic lesions, which give rise to CNVs [4, 5]. Regardless of the mechanism behind their creation, CNVs may delete tumor suppressor genes, or produce multiple copies of oncogenic ones, increasing the tumor fitness [6, 7]. The gain in fitness follows tumor evolution models, and recent work has linked copy number changes to immune evasion in ovarian cancer [8, 9]. While traditional multi-megabase amplification and deletions are an intuitive driver of cancer evolution, the same processes driving copy number changes also create focal structural variants (SVs), which contribute to tumor heterogeneity and plasticity [10].

Two such SVs, breakage fusion bridges (BFBs) and extrachromosomal circular DNA (ecDNA), exhibit an extremely high average copy number and highly variable copy number between cells [11, 12]. These SVs, through their high count and highly variable composition, drive intratumoral heterogeneity and allow tumors to adapt rapidly to overcome treatments and immune surveillance [10, 13].

In both cases, the adaptability conferred on tumors by the SVs make them an important topic of research. Since the SVs can have highly variable copy numbers, even within cells of the same phylogeny, it is important to study these SVs on a single-cell level. The tools best suited to these studies, single-cell whole genome sequencing (scWGS), can be distinguished into three categories: PCR-, isothermal amplification-, and transposase-based [14]. Polymerase chain reaction (PCR) and isothermal amplification-based scWGS both rely on degenerate oligonucleotide primers (DOP) to randomly prime and amplify the genome [15–17]. Due to the reliance on primer binding and uniform amplification of genomic DNA, these two methods suffer from allelic dropout or uneven coverage of the genome, requiring high sequencing depth to reconstruct the copy number changes [18, 19].

Direct Library Preparation Plus (DLP+) is a Tn5 transposase-based scWGS method that uses a robotic dispensing system to spot and lyse cells in nanoliter-scale wells of a 5184-well chip. The genomic DNA is converted into sequencing libraries in a sequential procedure that starts with simultaneous genomic fragmentation and library adapter ligation facilitated by the Tn5 transposase. Following tagmentation, the Tn5 enzyme is deactivated and per-cell barcodes are added to the genomic fragments via PCR amplification. Unlike DOP- or isothermal amplification-based methods, the Tn5 activity is randomly distributed across the genome, enabling accurate copy number recovery with shallow sequencing depth [20]. Furthermore, since library adapters are directly ligated to the genomic DNA, each pair of unique reads corresponds to unique genomic fragments. However, due to the random adapter pairings on DNA fragments, DLP+ has a maximum possible coverage of half the cell’s overall ploidy [14].

Since Tn5 tagmentation is relatively unbiased, the probability of sequencing a given fragment is proportional to the copy number state of the genomic region from which this fragment originated. As a result, the ratio of sequencing depth across genomic regions can be used to recreate the original copy number profile of a cell. There is a large family of existing tools developed for bulk whole genome sequencing (WGS), which take advantage of this property to segment the genome into regions with similar sequencing read depth and assign copy number. Examples of these tools use traditional Haar wavelet transforms (CoNVEX, and HaarSeg), hidden Markov models (CNVKit, HMMCopy), and recursive splitting (Circular Binary Segmentation) [21–24].

Originally, these tools were designed with microarray and bulk WGS data in mind, with some of them (HMMCopy and SCOPE) optimized for scWGS. HMMCopy relies on a hidden Markov model to identify the transition point between copy number states [25]. Since HMMCopy is limited to maximum copy number 15, it is unable to call copy numbers accurately for high copy SVs such as BFBs and ecDNA. SCOPE on the other hand fits a hierarchical model to the data, adjusting its copy number fitting for per-cell variations [26]. Since these tools rely only on binned read counts, they are unable to detect WGD events, as the read depth ratio between genomic regions is the same in WGD or regular cells.

CHISEL and Alleloscope both leverage single nucleotide polymorphism (SNP)-based phasing to estimate the cell’s average ploidy, solving the WGD non-identifiability problem [27, 28]. However, since both tools rely on phasing, they need higher depth than DLP+ can provide to accurately call copy number, and they cannot detect perfect WGD where the ratio between alleles at heterozygous SNPs remain one-to-one.

Two tools have taken advantage of the DLP+’s unique properties to solve the WGD problem, both using a careful preprocessing workflow to ensure that all reads are from unique fragments. scAbsolute, released in 2024, requires one bam file per cell and compares the overall read depth with local read density to estimate true copy number. It also relies on a hidden Markov model to identify copy number breakpoints, but uses an expectation maximization algorithm to fit the final copy number [29]. The most recently published tool, ASCENT, performs end-to-end alignment, copy number calling and phasing, and compares overlapping read statistics to the cell’s sequenced depth, using higher than expected overlapping read counts to identify cells with duplicated genomes [30]. Notably, ASCENT removes duplicate reads missed by standard deduplication workflows by filtering out any read that starts or ends within 1 nucleotide of another read.

Here, we present Songbird, a Haar wavelet based, absolute ploidy-sensitive copy number caller, which can be adapted to other Tn5-based WGS methods (Fig. 1). Songbird, minimally, requires the start and end coordinates of properly paired fragments and fits unbalanced Haar wavelets (asymmetric in amplitude and node distance) to sensitively identify the breakpoints in the genome [31]. Next, in order to estimate the cell’s mean ploidy (up to mean ploidy 6, which includes WGD), Songbird takes advantage of sequencing fragment geometry by recursively comparing read density upstream of, and immediately adjacent to each sequenced fragment, effectively regressing out per-cell quality effects. Finally, Songbird uses a likelihood ratio maximization algorithm to fit the true copy number given fragment count in the segmented genomic regions and the estimate of cell’s mean ploidy based on fragment geometry. To benchmark Songbird, we separately test the segmentation (Fig. 2) and raw ploidy estimation (Fig. 3) modules of the tool against other scWGS algorithms before evaluating the tool as a whole (Fig. 4).

In testing against simulated scWGS data, existing DLP+ datasets, and experimentally-designed ploidy ladders, we show that Songbird accurately captures high copy number events and estimates ploidy better than existing tools. In addition, Songbird is compatible with data aligned to hg19, hg38, and hs.1 versions of human genome.

## Materials and methods

### Patient information

Endoscopic and surgical samples were collected at the Cambridge University Hospitals NHS Trust (Addenbrooke’s Hospital) from esophageal adenocarcinoma patients undergoing cancer resection. The study was approved by the Institutional Ethics Committees (REC 07/H0305/52 and 10/H0305/1), and all subjects gave individual informed consent.

### Single-cell DNA sequencing

#### Tissue culture

The immortalized hTERT cell lines were derived from a male lymphoblastoid cell line. The hTERT and HEK293T cell lines were cultured at 37$^{\circ }$C in 5% CO_2_, and grown in RPMI-1640 or DMEM, respectively. Both media are supplemented with 2mM l-glutamine and 10% Fetal Bovine Serum. Cells were passaged every 2–3 days after achieving ~90% confluency. To passage, the cells were washed with 1× phosphate buffered saline, detached using 0.05% trypsin and reseeded at 1:5 split, and cell passage number was recorded.

#### DLP+ sequencing and ploidy ladder generation

DLP+ was performed in keeping with previously published protocols [20]. In order to create the sequencing ladder, a mixture of cells were placed into each well such that the total genomic content ranges from 2N to 10N. For each of the cell mixtures (1×,2×,3×,4× hTERT, 1× HEK293T, 1× hTERT and 1× HEK293T, 2× hTERT and 1× HEK293T, 1× hTERT and 2× HEK293T, and 2× hTERT and 2× HEK293T), we spotted 100 wells. We chose hTERT cells as a diploid control used in previous studies and HEK293T as readily available cell line with highly diverse aneuploidy states [20]. Prior to cell spotting, Illumina-compatible primers containing unique cell barcodes are spotted into a 72 × 72 array. After cell spotting, each well is loaded with QIAGEN protease, Viagen DirectPCR cell lysis buffer, and the cells are heat lysed at 50$^{\circ }$C for 60 min followed by buffer inactivation at 75$^{\circ }$C, 15 min. The tagmentation buffer is then added and incubated at 55$^{\circ }$C for 10 min. The Tn5 reaction was stopped by neutralization via Qiagen Protease at 50$^{\circ }$C for 10 min followed by an additional heat inactivation of the protease (70$^{\circ }$C, 15 min). After deactivation of the Tn5 enzyme, the samples underwent 11 cycles of PCR and the resulting amplified DNA were pooled from the individual wells and sequenced on an Illumina NovaSeq 6000 S4 flow cell to produce 150 bp paired end reads.

### Data preprocessing

The ploidy ladder and the esophageal adenocarcinoma sample were processed using the mondrian pipeline (https://github.com/mondrian-scwgs/mondrian); reads were trimmed using TrimGalore v0.6.6, and quality checked with FASTQC v0.11.9. Trimmed reads were then assigned to a read group based on the trimmed adapter sequence and aligned to the GRCh38 genome with decoy and alternative contigs and screened for contamination using FastQScreen v0.14.0. The per cell bam files were filtered to retain high quality (phred score >30), primary alignments mapped to the 22 autosomes and 2 sex chromosomes. The filtered bam is deduplicated using samtools (v1.16) fixmate and markdup functions before using bedtools (v2.30.0) bamtobed to generate the bedpe file that identifies genomic regions spanned by each fragment.

To prepare the SNV data for Alleloscope, we followed the tutorial provided on their github repository (https://github.com/seasoncloud/Alleloscope/tree/main/samples/SNU601/scDNA). This involved producing phased SNV calls from the bulk bam, converting the calls to a cell-by-position matrix using Vartrix (https://github.com/10XGenomics/vartrix), and generating a counts matrix in the same format. Alleloscope, SCOPE, and scAbsolute were run in line with their respective documentation. HMMCopy was run in a small optimization loop based on the mondrian pipeline. This involves multiplying the GC and mappability corrected read density against a series of multipliers, which will convert the read density into integer states. The multiplier value that minimizes the number of half copy calls (i.e. copy numbers 1.5, 2.5, 3.5, etc.) is retained as the true multiplier. For the ploidy ladder, ASCENT was run without a normal reference, with a 10-kb bin size, and with multiple gamma parameters (0.5, 1, 2, 5, 10). For final evaluation, gammas 0.5, 0.5, 10, 5, and 10 were used for the 1× hTERT, 1× HEK293T, 2× hTERT, 1× hTERT 1× HEK293T, and 3× hTERT conditions, respectively as they had the best performance relative to the ground truth. For the CAM277 data, since there is no ground truth to evaluate against, we used a gamma of 10 as it seemed to perform better with higher ploidy cells in the ladder.

Fastq files for each cell from the Laks ladder, PEO1 ladder and DnTR-Seq ladder were aligned using bwa-mem v0.7.17-r1188 and filtered for properly paired reads with both ends aligned to the 22 autosomes and 2 sex chromosomes. The per-cell bam files were then processed identically to the ploidy ladder. Explicitly this involved deduplication using samtools (v1.16) fixmate and markdup before generating the bedpe with bedtools (v2.30.0) bamtobed. For the Laks and DnTR-Seq ladders, we marked same-orientation reads that start or end at the same coordinate as duplicates and only retained the longest fragment of the duplicate pair. Since the PEO1 ladder only had 35-nt reads in the publicly available Fastqs, and our mappability scores were derived for 50-mers, we marked same-orientation reads that start or end within 5 nucleotides of each other as duplicates and retained the longest fragment of the duplicate pair.

LIANTI fastqs were processed following their github repository (https://github.com/lh3/lianti). Notably this involved using seqtk and trim from adna (https://github.com/DReichLab/adna) to merge paired reads and extract read UMIs prior to aligning with bwa-mem. The resulting per-cell bams were deduplicated and converted into a bedpe as above, with the UMI information saved for each read. We used the UMI information in addition to the read coordinates to perform an intermediate deduplication of the sample, retaining the longest fragment in any set of fragments with the same UMI. For the final deduplication, fragments that started or ended within 10 nucleotides of another same orientation fragment were flagged as duplicates, and the longest in a set of duplicates were retained.

SmoothSeq PacBio long read fastqs were processed usingpbmm2 released by PacBio (https://github.com/PacificBiosciences/pbmm2) with the CCS preset. The per-cell bam files were then deduplicated as described above. Since the PacBio fragments are not paired end, we generated a bed file using bedtools instead of a bedpe. Final deduplication flagged fragments with the same orientation that started or ended within 200 nucleotides of another fragment, and we retained the longest fragment in any set of duplicates.

### Simulated read generation

We use *CINner* [32], an algorithm for modeling the evolution of chromosomal instability, to create simulated copy number profiles. Each simulation starts with 1000 diploid cells, and the cell division probabilities are calibrated such that the total population size remains constant on average. Each cell division contains a missegregation of a randomly selected chromosome with probability $p_\mathrm{misseg}\in \lbrace 0.02,0.05,0.08,0.2\rbrace$. The copy number profiles observed after an average of 300 generations are then recorded.

We then simulate cell- and bin-specific fragment counts. For a given cell, let $\lbrace n_j,g_j\rbrace$ be the simulated copy number and GC content of bin *j*. We assume a total of $r=4\times 10^6$ reads per cell, following a Gamma distribution per bin with scale *σ =0.02* (approximated from previous DLP+ data). We simulate read count $r_j$ for bin *j* as


\begin{align*}n^{\prime }_j&=n_j\cdot g_j,\\n^{\prime \prime }_j&\sim \mathrm{Gamma}\left(\mathrm{shape}=\frac{n^{\prime }_j}{\sigma },\mathrm{scale}=\sigma \right),\\\lbrace r_j\rbrace _{j=1,2,\dots }&\sim \mathrm{Multinomial}\left(\mathrm{size}=r,\mathrm{probabilities}=\frac{n^{\prime \prime }_j}{\sum _j|n^{\prime \prime }_j|}\right).\end{align*}

The inferred copy numbers based on $\lbrace r_j\rbrace$ are then compared against ground-truth copy numbers $\lbrace n_j\rbrace$ to determine accuracy.

### Ground truth copy number estimation

The ground truth for the ploidy ladder was generated using ASCAT v3.2.0. Since ASCAT uses the frequency ratio between the heterozygous SNP, we constructed pseudobulk samples by merging all 100 cells per ladder condition into a single bam file. This assumes that all 100 cells in each condition are clonal. For the accurate copy number fit, ASCAT requires successful identification of heterozygous SNPs. In the absence of reference samples, any SNP with B-allele frequency (BAF) between 0.1 and 0.9 can be assumed to be heterozygous. However, since the samples are clonal, the heterozygous SNPs in the loss of heterozygosity (LoH) regions cannot be identified using this approach as all SNPs (heterozygous and homozygous) would have BAF close to 1. Therefore, in regions with suspected LoH, we randomly selected SNPs (at frequency similar to the genomic regions without LOH) and defined them as heterozygous and assigned a BAF of 1 allowing for CN calling. Next, ASCAT segments the genome using the BAF values of heterozygous SNPs and the final copy number is fitted along the spans using reads corrected for GC and mappability bias using default ASCAT settings. ASCAT was only run on the 1× hTERT and 1× HEK293T cell condition, as the multiplet conditions (2×, 3×, 4× hTERT) would collapse to the singlet copy number due to identical BAFs. Additionally, the mixed multiplet conditions (i.e. 1× hTERT + 1× HEK293T) cannot be used in ASCAT analysis as it contains a mixture of genomes from two individuals with different sets of SNPs what would produce inaccurate BAF values. As a result, the ground truth CN profile for higher ploidy states are a summation of the 1× hTERT and 1× HEK293T profile.

We followed the same procedure for the CAM277 organoid, but a significant amount of the sample ($\sim 40\%$) had LoH. As a result, ASCAT was unable to call BAF frequencies in large parts of the genome. In order to produce a final copy number profile, as before, we simulated SNVs in the LoH regions similar to the ladder, but due to the much higher proportion of LoH in the genome, these ground truth copy number calls are less confident.

### Songbird

Songbird is composed of four parts—breakpoint identification, ploidy estimation, subclone/whole genome duplication detection, and finally integer fitting.

#### Copy number breakpoint identification

We use QDNASeq to load in reads from the bam file, count the number of reads in the predefined genomic bins [default = 500 000 basepairs (bp)], and perform GC and mappability correction per cell following QDNASeq recommendations [33]. These corrections are run automatically during data loading from the bam files. If the user does not provide a bam file, the binned counts are aggregated from the bedpe file and subsequently GC+ Map corrected by QDNAseq in the same manner. In order to identify breakpoints where the true copy number state changes in the GC- and mappability-corrected read count space, we use the unbalanced Haar wavelet transform [31]. The main difference between traditional Haar wavelets and unbalanced Haar wavelets is that the former can only propose a breakpoint in the middle of a span, while the latter can propose a breakpoint at the most likely location, not just in the middle. This flexibility allows it to correctly identify breakpoints in genomic segments where the copy number state change is not in the middle of the segment. The algorithm then scores the fit of the split and recurses on two new spans before and after the split. The recursion completes when remaining spans have length 1 and breakpoints with support greater than a threshold are retained. In our data, we treat the entire genome as the time series data, ordered by chromosome and coordinate, and the threshold is set to the median absolute deviation of the read depth, as recommended by the original paper [31].

Since the unbalanced Haar algorithm can split the data at any point in a given span, it can erroneously fit a span with no true breakpoints by proposing a breakpoint at the end or the start of the span that happens to fit a noisy observation. These create segmented spans that we refer to as singlets since they do not share the segmented state with either the previous or following bin. While most singlets are likely erroneous, singlets derived from areas with high CNV may reflect true focal amplifications such as ecDNA or BFB. Therefore Songbird replaces singlets below the 90th percentile read depth with the previous bin’s segmented value, and replaces singlets above the 90th percentile with their bin’s read depth.

#### Ploidy estimation

Accurate ploidy estimation relies on strict removal of any missed duplicate fragments, and the knowledge about the minimum and maximum fragment lengths ($L_{\mathrm{ min}}$ and $L_{\mathrm{ max}}$, respectively). In order to achieve this, from the subsequent analysis we exclude the following:

- Shorter fragments from pairs of fragments that share a start or an end coordinate, and their orientation (we retain the longer fragments from the pair);
- Fragments shorter than a user-defined $L_{\mathrm{ min}}$;
- Fragments longer than the 99th percentile of fragment lengths ($L_{\mathrm{ max}}$);
- Fragments from bins with <90*%* mappability, bins which are <90*%* assembled, or bins which are in the ENCODE exclusion list [34].

Furthermore, we only use the 5’ ends of the reads to define the start and end of each fragment, and remove the first 10 nucleotides from each fragment to correct for the nucleotide duplication generated by Tn5 tagmentation between adjacent fragments. This stringent filtering omits ~45*%* of the genome, but ensures that we are only working with highly confident set of unique fragments generated by DLP+.

After filtering, we know that the remaining fragments are a subsample of the original *n* copies of genetic material, with the chance of observing a fragment equal to the probability of sequencing a read from one allele ($P_{\mathrm{ obs}}$) times the number of alleles, *n*. Since each fragment is derived from a distinct allele, if we observe a reference fragment, we know that there are only *n-1* alleles available to generate overlapping fragments, thus the chance of observing an overlapping fragment is $(n-1) P_{\mathrm{ obs}}$. Given the minimum fragment length, $L_{\mathrm{ min}}$, we know that fragments starting less than $L_{\mathrm{ min}}$ nucleotides prior to the starting coordinate of thereference fragment must be drawn from *n-1* alleles with a $(n-1) P_{\mathrm{ obs}}$ chance of being sequenced. In contrast, in a “non-overlapping window” located at least $L_{\mathrm{ max}}$ bp upstream of the start of thereference fragment, there is a chance of $n P_{\mathrm{ obs}}$ of observing a fragment. Since $P_{\mathrm{ obs}}$ is common to both chances of observing a fragment, the ratio of overlapping to upstream fragments counts depends only on *n*, allowing us to estimate the true copy number directly from observed fragment counts. However, given DLP+’s low coverage, the chance of observing overlapping fragments is low, so we treat every filtered fragment as a reference fragment, and add the upstream and overlapping fragment counts within each bin to get a reliable estimate of the overlapping versus upstream fragment ratio. This estimated ratio is averaged across all bins that pass the quality metrics mentioned above and used for the final calculation of *n*.

In practice, $P_{\mathrm{ obs}}$ is not perfectly uniform across the genome due to variations in genome accessibility and gDNA preservation. Despite this, Illumina paired end sequencing rarely sequences fragments larger than 1.5 kb, so the upstream and overlapping fragments are likely drawn from regions of the genome with similar accessibility and quality, so $P_{\mathrm{ obs}}$ is consistent over this short span. The number of overlapping and upstream fragments are summed per bin and the ratio calculated per bin. For final ploidy estimation, the ratio is averaged across all bins and copy number estimated from that final ratio. The exact implementation of our ploidy estimation algorithm is in Algorithm 1.

Due to the processes underlying genomic instability, cells with significant aneuploidy may contain short regions with high abundance of sequencing fragments including high frequency of fragments with the same start or end coordinates (e.g. focal amplifications or ecDNA). In these regions, the number of upstream and overlapping fragments observed will be significantly higher and potentially uninformative as the ratio between them would approach 1. Since the upstream and overlap counts are summed per bin and the other genomics regions have relatively low fragment counts, these high coverage regions can bias the bin’s ploidy estimate. As a result, we exclude reads outside of the middle 98th percentile range of overlap and upstream counts when estimating ploidy.


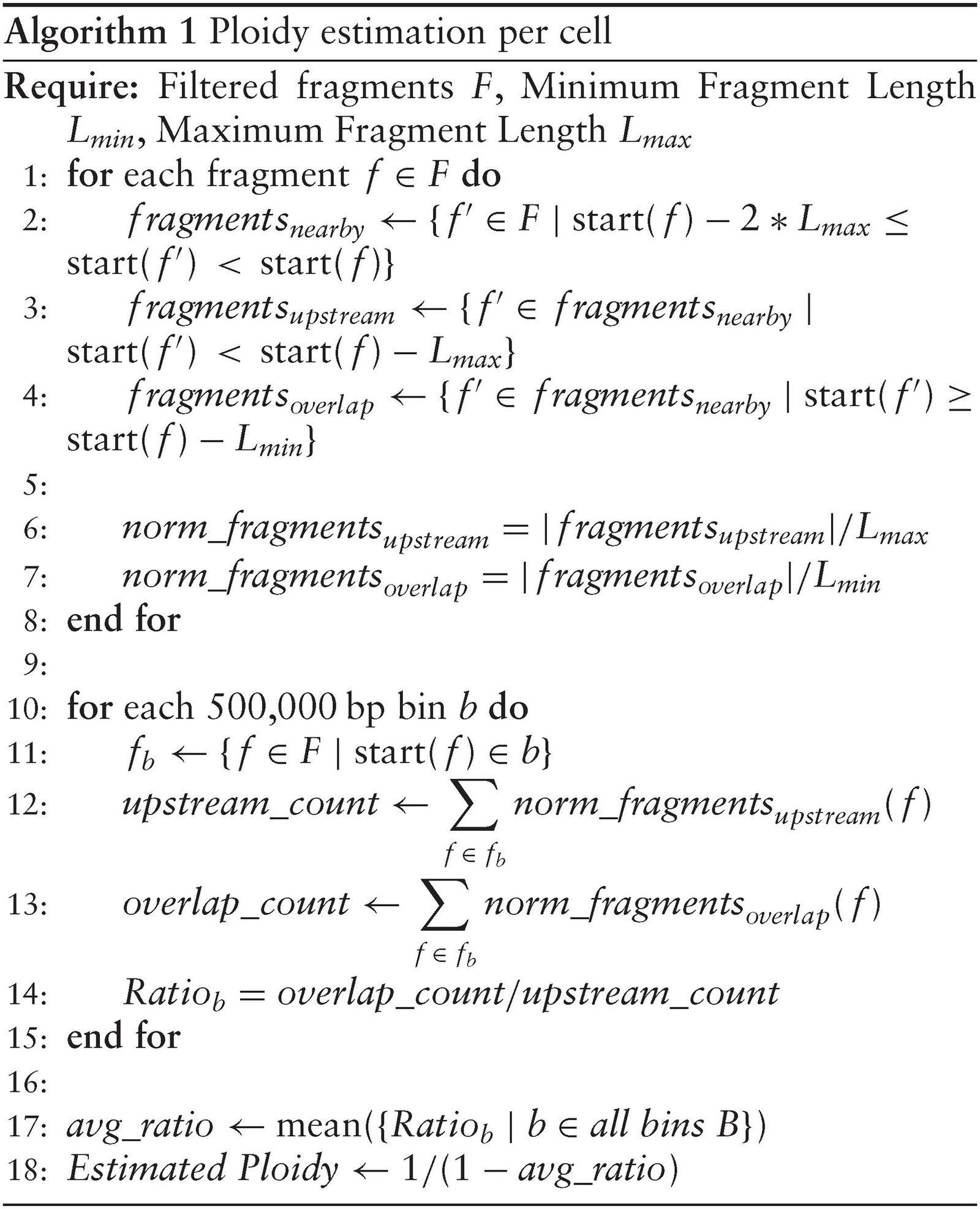


For sequencing methods with less complete duplicate fragment removal, such as PacBio, we exclude observed fragment starts close (within 100 nucleotides) to the reference fragment start. Additionally, the upstream window for long read sequencing could stretch into regions of the genome that were not similarly preserved or uncompacted as the reference region. In this case, $P_{\mathrm{ obs}}$ is no longer the same in the upstream and overlapping windows, so we have to enforce an upper limit on the fragment lengths, which in our limited experience is ~8 kb.

#### Subclone identification, ploidy correction, and whole genome duplication detection

To help correct the noisy ploidy estimates obtained in the prior step, we use information from all cells and identify subclones with similar copy number states followed by the calculation of the pooled ploidy estimates. Subclones are identified by clustering the cells using the positions of the copy number breakpoints between cells. In more detail, we find variable breakpoints (observed in at least 10% and at most 90% of cells) apply a box kernel to minimize the difference between breakpoints that are offset by one bin, and use the Phenograph algorithm to identify subclones [35].

In order to detect WGD, we try to find a set of cells within each subclone which has approximately twice the estimated ploidy as the rest of the subclone. Songbird does this by fitting a *k=2* K-means clustering model on estimated ploidy from cells with >50 000 reads within each subclone. If the two sub-clusters within each subclone are sufficiently compact (${\rm between\, cluster\, variance}/{\rm total\, variance} \le 0.7$) and the centroids are close to a 1:2 ratio, cells in the higher ploidy cluster are flagged as WGD. The estimated ploidies for WGD and non-WGD cells within each subclone are then averaged separately to get a corrected ploidy for the WGD and non-WGD cells.

#### Integer fitting

Since DLP+ fragments are derived from unique alleles, we know that the count of deduplicated reads sequenced in any genomic bin should be linearly proportional to the copy number in that same region. Therefore for each sequenced cell, there is a true scaling factor that maps the read depth at any genomic location to a true copy number. We refer to this true scaling factor as the reads per copy number (RpCN) value.

To estimate the RpCN, we use the cell’s estimated ploidy from the prior step. The most frequently observed read depth, the mode of the read depth distribution, should correspond to the most common copy number in the cell, approximated by the estimated ploidy. We estimate the mode by applying a Sheather–Jones kernel density estimator to the distribution of per-bin read depths using the read depth with the highest density.

Because the ploidy estimate is noisy, we propose a set of candidate integer ploidies that the modal read depth could correspond to, ranging from 0.75 to 1.5 times the estimated ploidy. This set of proposed ploidies implies a set of RpCN by dividing the modal read depth by the proposed ploidies. The candidate RpCN values for each proposed ploidy are tuned by maximizing the log likelihood ratio of the generative model against a null—the same scoring function used for final evaluation. The tuned value for each ploidy is retained as $\mathrm{ RpCN}_p^{*}$. Since high copy number, focal amplifications are noisy and can bias our scoring, all scoring is not performed on bins above the 90th percentile segmented state.

Each candidate RpCN is scored using a generative model of read depth. In this model, the read depth of each bin *b* is normally distributed with mean equal to the bin’s implied integer copy number ($\mathrm{ round}({\rm read\, depth} / \mathrm{ RpCN})$) times the RpCN scaling factor. The variance for each distribution is the mean squared deviation between the unsegmented ($r_n$) and segmented ($b_n$) read depths across all bins:


\begin{align*}
\sigma ^2 = n_{\mathrm{ bins}}^{-1}\, \sum _{n}( r_n - b_n)^2.
\end{align*}

Since the variance is fixed, models proposed by higher candidate ploidies will always fit better than lower candidate ploidies. As a result, we score each RpCN by the log likelihood ratio of the generative model against a null distribution. This null model assumes that reads are equally likely within the cell’s observed dynamic range [approximated by Uniform(10th, 90th percentile read depth)]. The optimized RpCN, $RpCN_p^{*}$, with the highest log-likelihood ratio between the segmented fit and the null fit is selected and copy numbers assigned accordingly. If there is an estimated ploidy for the cell, the score for each RpCN is given a gaussian penalty based on the cell’s average copy number deviation from the estimated ploidy. Algorithms (2) and (3) provide the implementation and scoring functions, respectively.


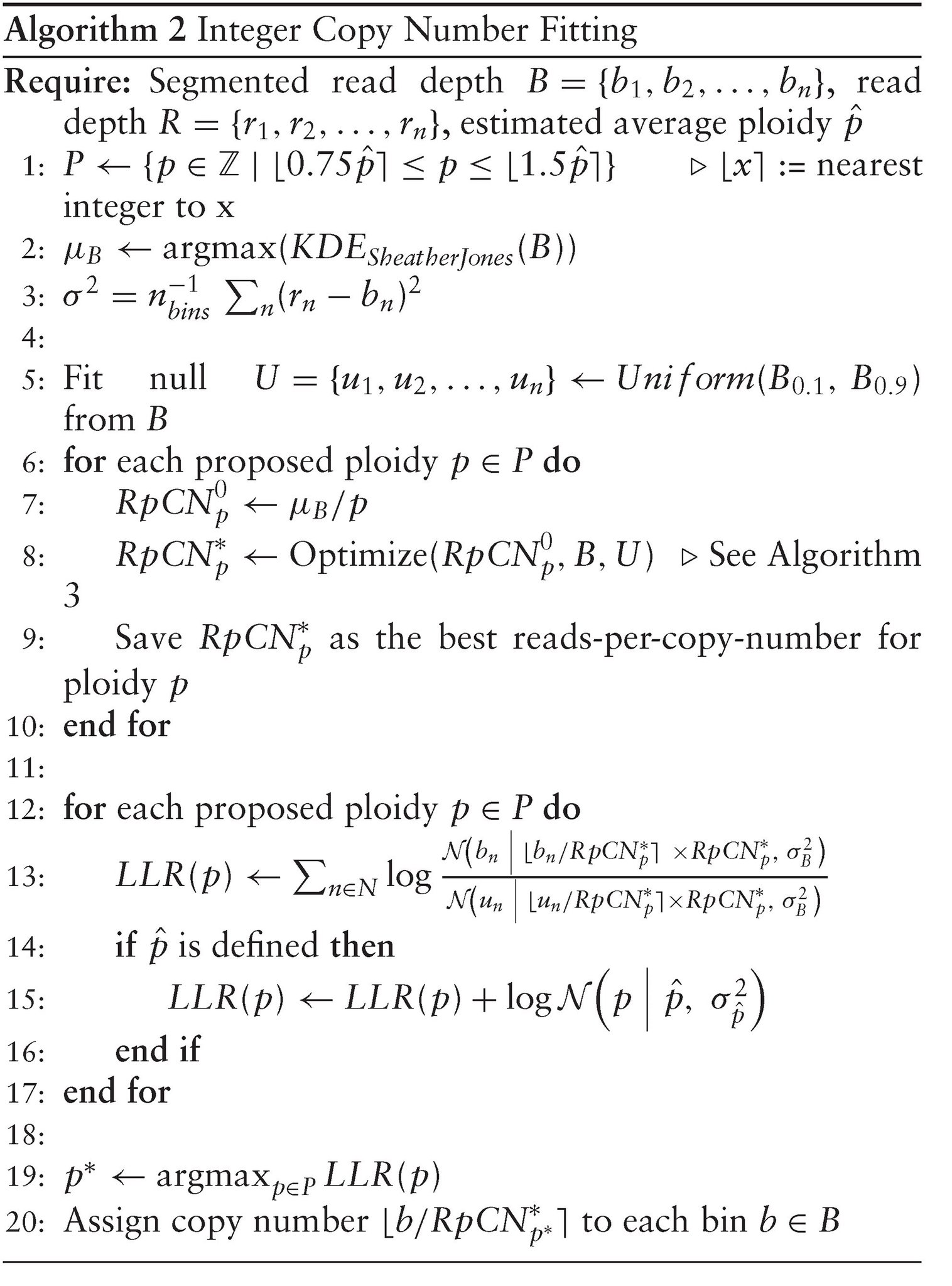



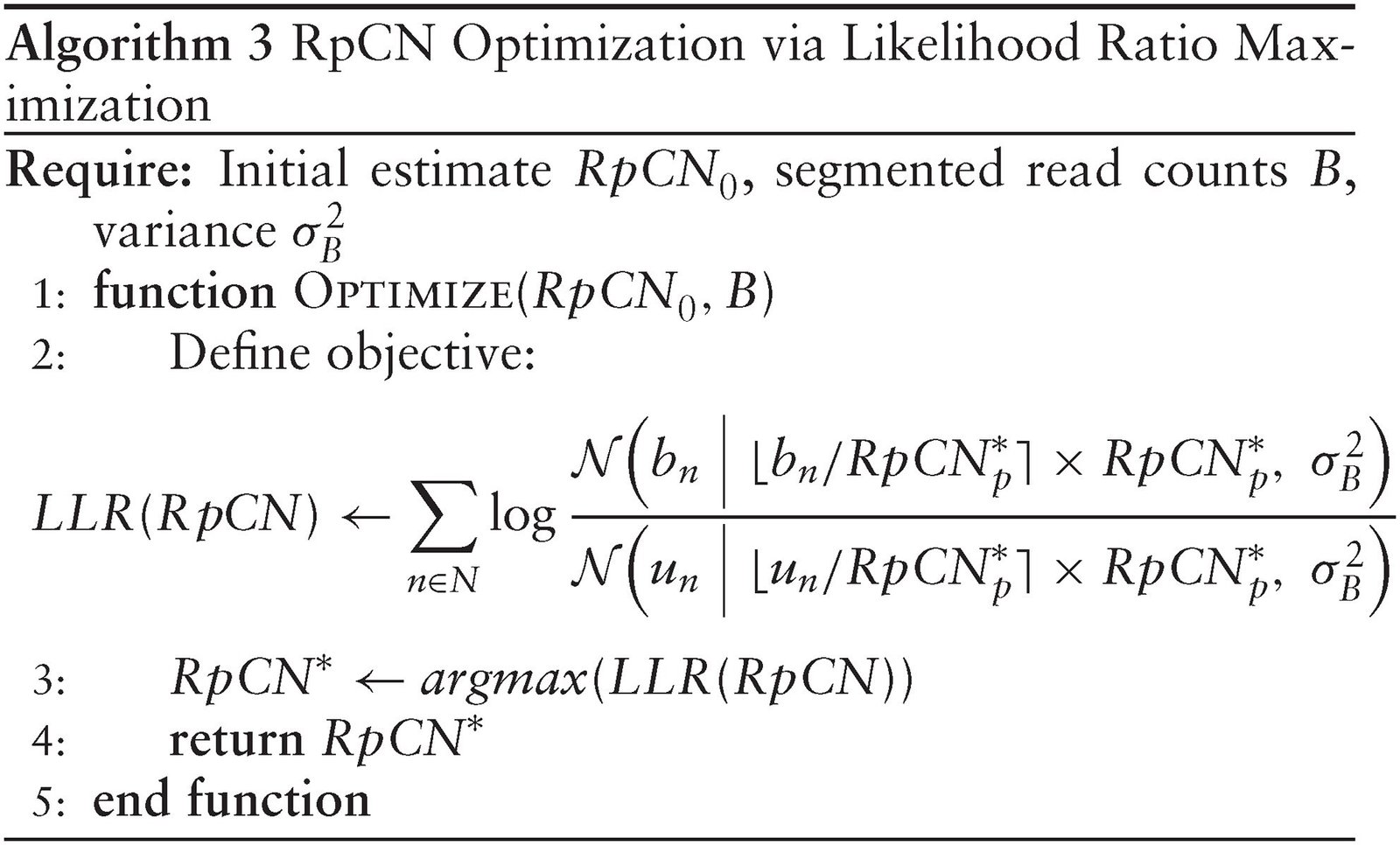


### Segmentation performance comparison

To quantify segmentation performance in the simulated data, we compared Songbird’s implementation of unbalanced Haar against traditional unbalanced Haar, CoNVEX, HaarSeg, HMMCopy, and circular binary segmentation. All tools were implemented using default parameters, except HMMCopy, which was run as described above. CoNVEX’s segmentation algorithm was re-implemented as described in the CoNVEX paper using the R package wavethresh v4.7.2 since there are no publicly available implementations. Performance was assessed using two metrics, the Jaccard Index and Wasserstein Distance. Jaccard Index compared the true breakpoints in either the simulated or ASCAT processed data to the set of breakpoints proposed by each tool. For the Wasserstein distance, we scaled the copy number by the RpCN for each cell prior to calculating the distance since the ground truth copy number is not on the same scale (integer, copy number per bin) as the segmented read depth (real, reads per bin). The RpCN value is prespecified in the simulated data, but for the ploidy ladder, the RpCN was calculated by dividing each cell’s binned read counts by their respective ground truth copy number and averaged across all bins.

### Copy number estimation comparison

To compare each tool’s ability to recover the true average ploidy of each element of the copy number ladder, we averaged the estimated copy number profile for each cell across all tools tested. When comparing the actual copy number profile against the ASCAT ground truths, we used the root mean squared error (RMSE) between the estimated copy number profile and the ground truth profile. In the case of the organoid data where there is significant heterogeneity in each cell’s copy number, a singular ground truth copy number profile is not a valid comparison. Therefore, we measured copy number accuracy by calculating the Shapiro–Wilk test for normality against the distribution of reads assigned to each copy number. The quantile–quantile comparison was calculated similarly—reads assigned to each copy number by each tool were fit to a normal distribution using the mean and standard deviation of the reads. The normal distributions were then used to propose an expected quantile for each read value and compared to the observed quantile.

## Results

### Tn5-based scDNA-seq libraries contain artifactual reads that bias the frequency of read overlaps

Since DLP+ generates fragments derived from unique copies of the genome, simply observing the patterns of overlapping fragments would be an intuitive way to call the copy number profile. Ostensibly the maximum fragment depth at any location would indicate the true copy number state of that region of the genome. We observed two difficulties with this approach. First, the shallow nature of DLP+ sequencing meant the maximum fragment depth significantly underestimated the true copy number of high copy number regions. Second, even after excluding problematic genomic regions such as those found on the ENCODE exclusion list [34] and only retaining regions with high mappability scores, we observed a significant number of regions in the genome where the overlap state incremented in steps of two or more, indicating multiple reads starting or ending at the same coordinates. When compared to simulation of random read generation, we observe a significantly higher proportion of two state transitions than expected (Fig. 1A). Since we removed classically defined (i.e. the same start and end coordinates) duplicate fragments prior to our bedpe file generation, this indicated that there were a large number of fragments that started or ended at the same position but were of different lengths. Furthermore, our data were generated with a diploid, hTERT overexpressing cell line with no CNV outside of chromosome 20 (Supplementary Fig. S1). Therefore the maximum expected depth should not exceed 2, which is clearly violated by these fragment pairs. Interestingly, a large majority of these pairs had identical genomic orientations, outnumbering pairs with opposite orientations in a nearly 10:1 ratio (Fig. 1B).

**Figure 1.**
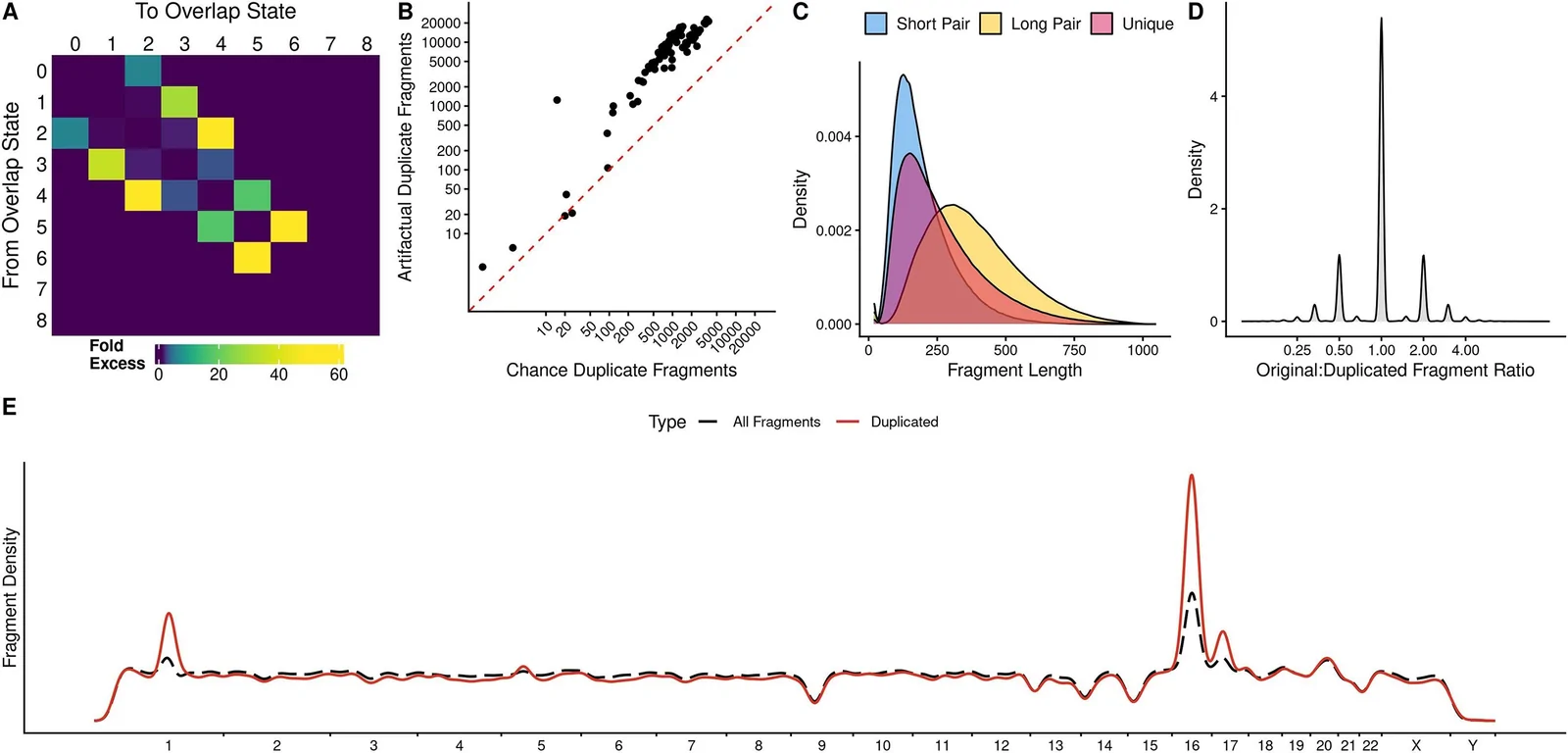
Tn5-based scDNA-seq sequencing generates artifactual sequencing fragments. (**A**) Heatmap of the fold excess in observed transitions in 100 hTERT cells versus a simulation. An excess of 0*→*2 transitions indicates that there are more reads starting at the same coordinate than expected by random read placement. (**B**) Scatterplot comparing the number of artifactual duplicate fragments (same start or end and same orientation) with the number of chance fragments (same start or end, but different orientations) in 100 diploid hTERT cells. (**C**) Fragment length distributions for the shorter (blue) and longer (yellow) member of the artifactual fragment pairs compared to the unique fragment length distribution (red). (**D**) Ratio of longer (original) to shorter (duplicated) fragment counts in the original bam prior to standard duplicate (same start, end and orientation) read removal. (**E**) Density across the genome for artifactual and unique fragments calculated over 100 diploid hTERT cells.

After further investigation of the fragment pairs, we observe that the shorter fragment in these potentially artifactual pairs were shorter than a typical fragment, while the longer fragments were longer (Fig. 1C). Further investigation of these pairs prior to duplicate fragment removal showed that these fragments pairs are overwhelmingly amplified in a 1:1 or 1:2 ratio, hinting that the process that produces these fragments occurs within or prior to the first cycle of PCR amplification (Fig. 1D). While there are some regions where these fragments are more common than others, the density of these potentially artifactual sequenced fragments largely mirrors the distribution of all fragments generated in these cells (Fig. 1E). Furthermore, any deviations between all fragment and artifactual fragment density seems to be a function of genome version as they shift in location depending on the genome that the data is aligned to (Supplementary Fig. S2). Since they are a minority of the total fragment count (*< *1%) but constitute a significant proportion of overlapping fragments, we discard the shorter fragment from each artifactual pair.

### Songbird accurately recalls copy number breakpoints in simulated and real scDNA-seq data

Having characterized the artifactual sequencing reads, we established the Songbird algorithm to calculate the absolute copy number states of each cell (the “Materials and methods” section). The first step focuses on the identification of breakpoints where the copy number state changes within the same chromosome. In order to validate Songbird’s breakpoint detection sensitivity we simulated reads from 4000 cells using CINner [32] with increasing rates of chromosomal instability (Fig. 2A), and fit Songbird, HMMCopy, HaarSeg, CoNVEX, Circular Binary Segmentation, and unfiltered unbalanced Haar algorithms to each cell. Breakpoint recovery was quantified by calculating the Jaccard index between the coordinates of the estimated and true breakpoints. Since the Jaccard index is intolerant of even one-bin offsets, all methods had relatively low scores, but Songbird outperformed HMMCopy and other Haar wavelet methods and was comparable to Circular Binary Segmentation (Fig. 2B). For a less strict comparison of copy number profile, we scaled the segmented values by the reads per copy scaling factor for each simulated cell and used the Wasserstein distance to compare the segmented reads to the true copy number profile. Since the segmented states are scaled to have the same magnitude as the copy number profile, a Wasserstein distance of 0.5 implies an approximate average error of 0.5 copies. Given this, all tools except HMMCopy performed well in recovering the overall copy number profile of the simulated data (Fig. 2C). All together, in the simulated data, Songbird’s implementation of the unbalanced Haar algorithm indicate an improvement over existing Haar wavelet methods, and is comparable to Circular Binary Segmentation with slightly better performance in samples with high copy number diversity.

**Figure 2.**
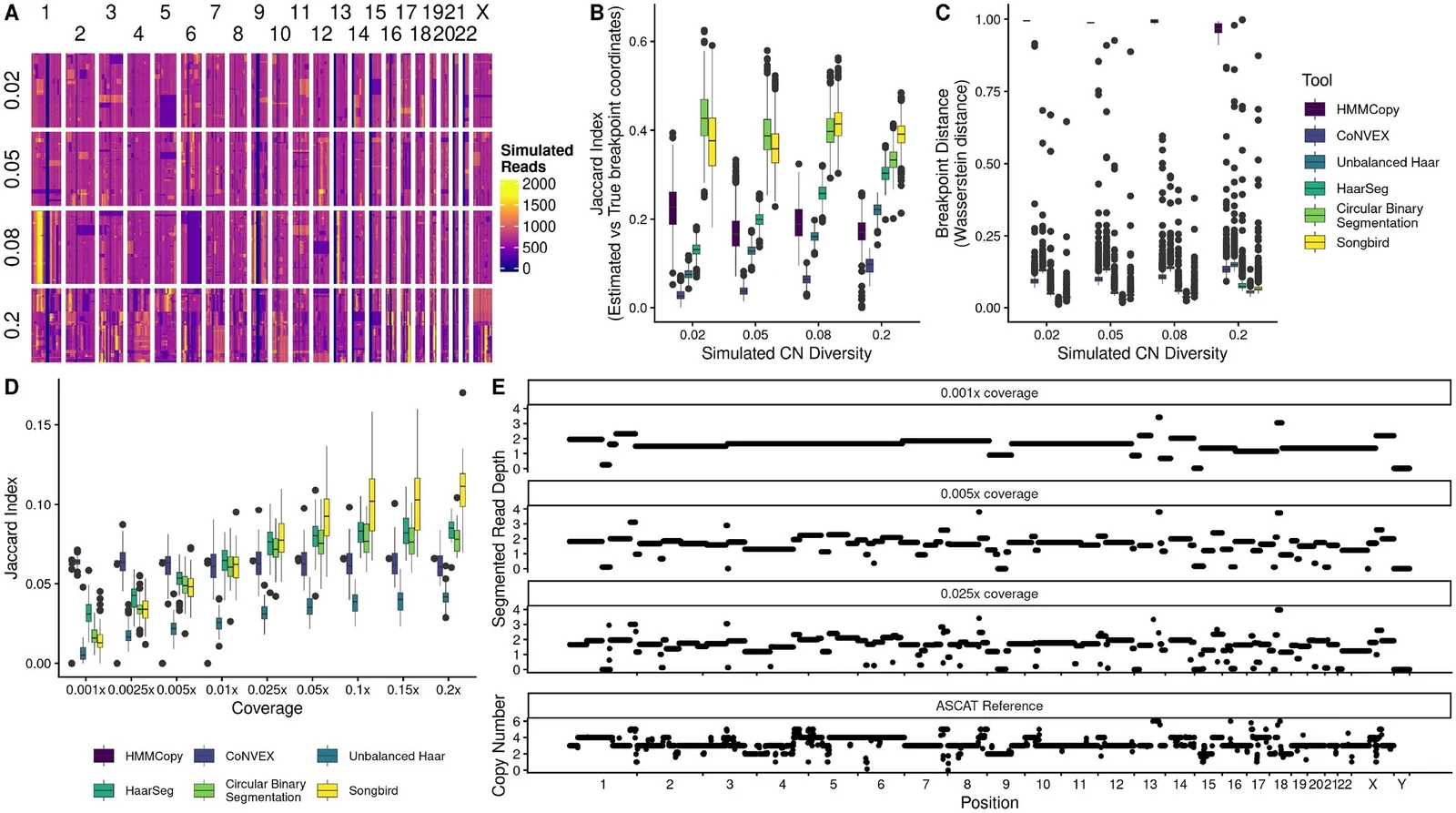
Songbird accurately identifies copy number breakpoints in scDNA-seq data. (**A**) Heatmap of simulated read depth in reads generated by CINner with increasing missegregation rate parameters. (**B**) Jaccard Index scoring the recovery of the simulated breakpoints in the simulated data. (**C**) Wasserstein distance between the segmented state and true copy number in the simulated data. The segmented states were scaled by each cell’s true RpCN ratio prior to distance calculation. (**D**) Jaccard Index scoring the recovery of true breakpoints in 100 HEK293T cells. Ground truth breakpoint coordinates were identified via bulk copy number calling by ASCAT. (**E**) Qualitative comparison of the segmented state across downsamplings for 1 HEK293T cell versus the true copy number profile generated by ASCAT.

In order to test Songbird’s segmentation algorithm in real data, we generated a ploidy ladder by spotting a DLP+ nanowell array with combinations of hTERT and HEK cells such that the expected ploidy per well ranges from 2 to 10. Since the perfectly diploid hTERT cells should have no breakpoints, we compared the performance of the various segmentation algorithms on the single HEK cell condition only, using the copy number profile generated by ASCAT as a ground truth. Similar to the simulated data, the Jaccard index for recovered breakpoints is relatively low, but Songbird’s implementation of unbalanced Haar is able to recover the same or more true breakpoints than all existing tools down to 0.01× coverage per cell. Below 0.005× coverage, HMMCopy and CoNVEX’s implementation of Haar wavelets perform better with breakpoint identification. Both tools notably oversegment the data, so the consistent but low Jaccard score could be due to the high number of predicted breakpoints (Fig. 2D). For reference, 0.01× coverage corresponds to 100 000 properly paired fragments per cell assuming a 2 × 150 nucleotide read length (Fig. 2D). Furthermore, our unbalanced Haar implementation seems to be relatively stable, with even 0.01× coverage subsamples correlating highly (*> 0.9* Pearson correlation) with the original segmentation profile across the ploidy ladder (Supplementary Fig. S3A). Similar to the results in the simulated data, the Wasserstein distance is comparable between methods (except HMMCopy) with an *∼ 0.2* average copy number error (Supplementary Fig. S3B).

### Songbird accurately estimates total cell ploidy

In order to estimate cell ploidy, we take advantage of the fact that unique fragments generated in a DLP+ run are all derived exclusively from fragments of the original genome. Intuitively, this means that the true ploidy of a cell could be identified by the maximum overlapping fragment depth. Unfortunately, the DLP+ process is relatively shallow, and variable cell quality can easily confound simple pileup counting. For example a high coverage, high quality diploid cell could have a similar maximum overlap state to a low coverage, degraded, tetraploid cell. For notation’s sake, we define $p(\mathrm{ Obs})$ as the joint probability of recovering a fragment from a cell and sequencing it. Since the pooled steps are shared across all cells, $p(\mathrm{ Obs})$ is an observable metric which is proportional to each cell’s quality, notated as $p(\mathrm{ Recovery})$ (Fig. 3A).

**Figure 3.**
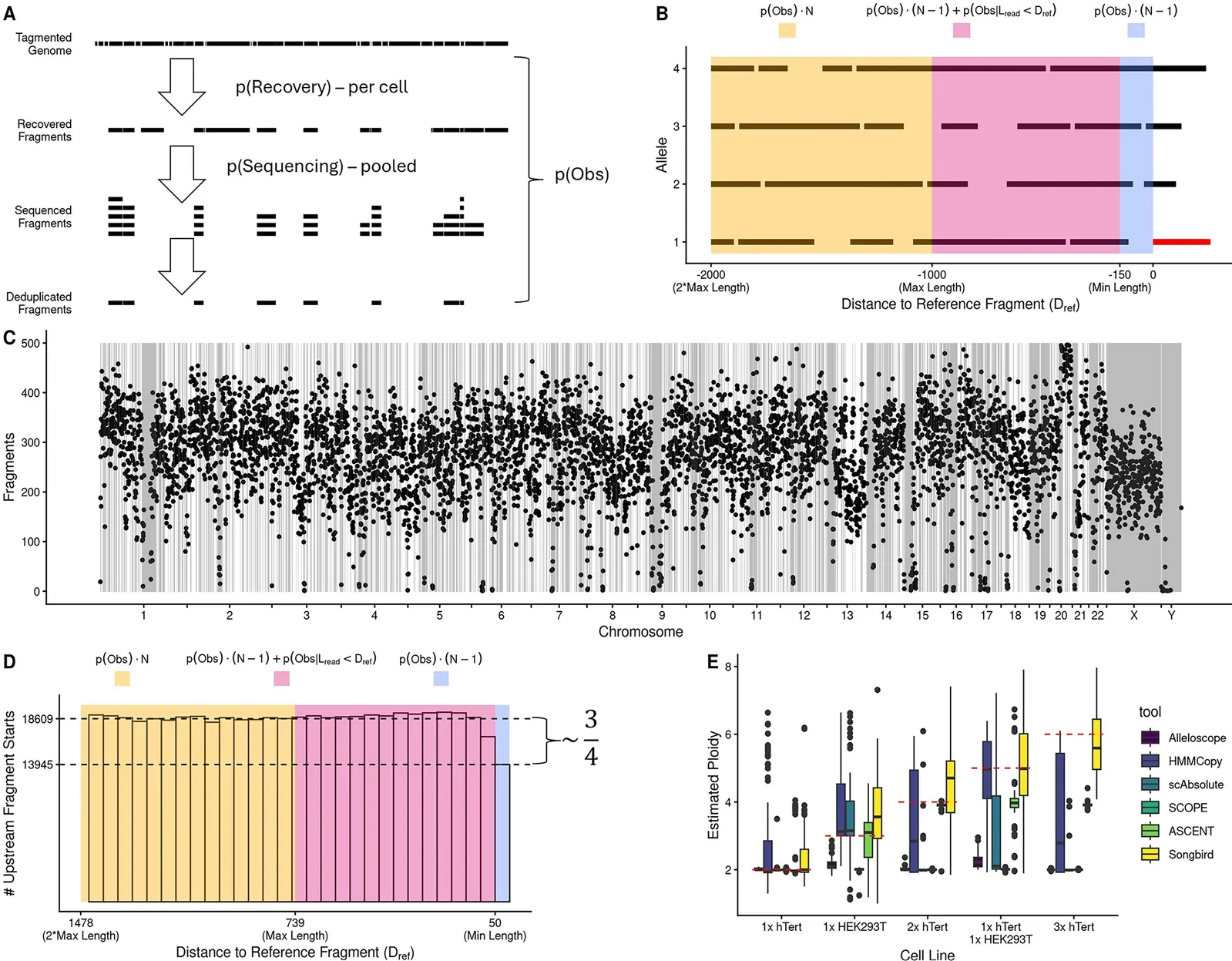
Estimation of mean cellular ploidy. (**A**) Cartoon of the read generative process. p(Recovery) is the probability of recovering a fragment from each cell and p(Sequencing) is the probability that the fragment gets sequenced once pooled. p(Obs) is the joint probability of recovering a read from each cell and sequencing after pooling. (**B**) Depiction of the ploidy estimation logic. Reads starting in the blue region only have *n-1* copies of the genome to draw from, while reads in the yellow upstream region have *n* copies of the genome to draw from. (**C**) GC + Mappability corrected reads for the 2× hTERT condition. Gray bars indicate regions of the genome excluded from ploidy estimation for quality purposes. (**D**) Read start counts upstream of all reads in the 2× hTERT cell in panel (C), aggregated into 50-bp bins. The colored windows mirror the ones in panel (B) with the maximum fragment length defined by the 99th percentile fragment length. The overlapping (*n-1*) read density divided by the upstream (*n*) read density produces a ratio that recovers the true ploidy (*n = 4*). (**E**) Average copy number for each of the ploidy ladder conditions across several tools.

Songbird accounts for $p(\mathrm{ Obs})$ by performing local fragment depth comparisons around each read. In essence, if we observe a reference fragment in a region with *n* copies of the genome, we know that there are only *n-1* copies left to draw overlapping fragments from. In practice this calculation is not straightforward, since a long reference fragment can be overlapped by multiple shorter fragments derived from the other alleles. To resolve this problem Songbird enforces a minimum fragment size cutoff $L_\mathrm{min}$. This enables us to look at a window of $L_{\mathrm{ min}}$ nucleotides upstream of our reference fragment and we know that the observed fragments starting in that window must come from different alleles and must overlap our reference fragment. If we look more than the longest fragment length, $L_{\mathrm{ max}}$, upstream of our reference fragment, we know that the fragments starting in that window do not overlap the reference fragment and are derived from *n* copies of the genome (Fig. 3B). To ensure that the overlapping fragments are only derived from unique alleles, Songbird excludes large regions of the genome due to assembly, mappability, or overlap with the Encode exclusion list (Fig. 3C).

Since the DNA quality and accessibility near a fragment is likely similar, the probability of recovering a fragment is likely common between the overlapping and upstream regions, removing the quality confounder. Songbird does this by dividing the density of fragments overlapping a fragment by the density of fragments upstream of the fragment. This noisy measurement is aggregated across all properly paired fragments and used to estimate ploidy (the “Materials and methods” section). With a sufficient number of reference fragments the ratio of overlapping to upstream read density approaches *(n-1)/n*, and the ploidy *n* of a cell can then be estimated as $n \approx 1/(1-\text{overlap density}/\text{upstream density})$ (Fig. 3D).

We validated Songbird’s ploidy estimation algorithm by testing Songbird’s initial ploidy estimates against other tools’ final copy number profile on our ploidy ladder (Fig. 3E). Since Songbird’s raw ploidy estimates are compared to the average of the final copy number called by other tools, Songbird’s estimations display lower precision than other tools. Despite that, the Songbird’s median ploidy is accurate up to copy number 6, and the accuracy of calls was independent of the version of human genome assembly (Supplementary Fig. S3C). Above copy number 6, Songbird struggled to accurately recover true ploidy, underestimating the copy number 7, 8, and 10 conditions. (Supplementary Fig. S3C). The reference hTERT cell lines, largely free of copy number alterations, highlighted the weakness of read depth only tools such as HMMCopy and SCOPE, which called the hTERT multiples as copy number 2. While relying on more information than just the read depth ratio, Alleloscope and scAbsolute seem to display a tendency toward calling diploid cells when segments at different copy number states are absent. Only ASCENT and Songbird were able to successfully call the hTERT 2× conditions.

Furthermore, Songbird’s ploidy estimator produces consistent ploidy estimates using smaller bins sizes (down to 50 kb, Supplementary Fig. S3D) and in high CNA (average ploidy *∼*5) cells down to 0.01× coverage, highlighting its robustness (Supplementary Fig. S3E). In a small subset of cells with predominantly high coverage, Songbird’s ploidy estimator overestimates mean ploidy—a potential artifact of S/G2-phase cells present in the samples. This can be most clearly observed in the 1× hTERT copy number profile (Supplementary Fig. S1).

To validate Songbird’s filtering and ploidy estimation process outside of our ploidy ladder, we tested Songbird’s ploidy estimator against previously published cell cycle and PEO1 ladders from the original DLP+ paper [20] and scAbsolute [29], respectively. In both cases Songbird accurately estimates the ploidy (Supplementary Fig. S4A and B).

Furthermore, Songbird’s ploidy estimator is directly extendable to DnTR-Seq [36], and shows promise with other Tn5 based scWGS methods such as LIANTI [37] and SmoothSeq [38] (Supplementary Fig. S4C–E). For both LIANTI and SmoothSeq, we removed reads that start or end within 10 (LIANTI) or 100 (SmoothSeq) nucleotides of another same-orientation read in order to get accurate ploidy estimates. Additionally, we limited the maximum fragment length for the SmoothSeq reads to 8 kb to make sure that $p(\mathrm{ Obs})$ is still locally comparable for each fragment. Even so, one-third of the SmoothSeq cells still had incomplete deduplication, producing erroneous ploidy estimates below zero.

### Songbird accurately recovers copy number profile

Since the count of DLP+ reads is linearly proportional to the bin’s copy number state, Songbird performs final copy number estimation by fitting a read generation model to the binned read counts for each cell. This model proposes a normal distribution for each bin that generates reads with mean equal to the true copy number multiplied by the RpCN scaling factor (see the “Materials and methods” section). The RpCN scaling factor is defined as the modal bins’ read depth divided by the estimated average ploidy. Since the ploidy estimate is noisy, we propose a set of true ploidies ranging from 0.75 to 1.5 times the estimated average ploidy, each of which produces their own read generation model. Since the variance is the same for each of the proposed ploidies, population variance of corrected reads versus segmented reads, a likelihood fit would trend toward picking the model with the highest proposed average ploidy (Fig. 4A and B). Therefore Songbird produces a null read distribution for each cell, and the best model has the highest likelihood ratio between the segmented and null data. Since the modal read depth is estimated via a kernel density estimator, we optimize the modal read depth for each proposed model prior to final evaluation (Fig. 4C and D). This produces a copy number profile, which closely resembles the ground truth as demonstrated with one HEK293T cell (Fig. 4E and F) and across the ploidy ladder (Fig. 4G), exceeding the accuracy of existing tools. Aside from Songbird, ASCENT notably produced a precise copy number reconstruction of the 1× and 2× hTERT cells, and HMMCopy performed well with the diverse copy number profiles present in the 1× HEK and 1× hTERT/1× HEK conditions.

**Figure 4.**
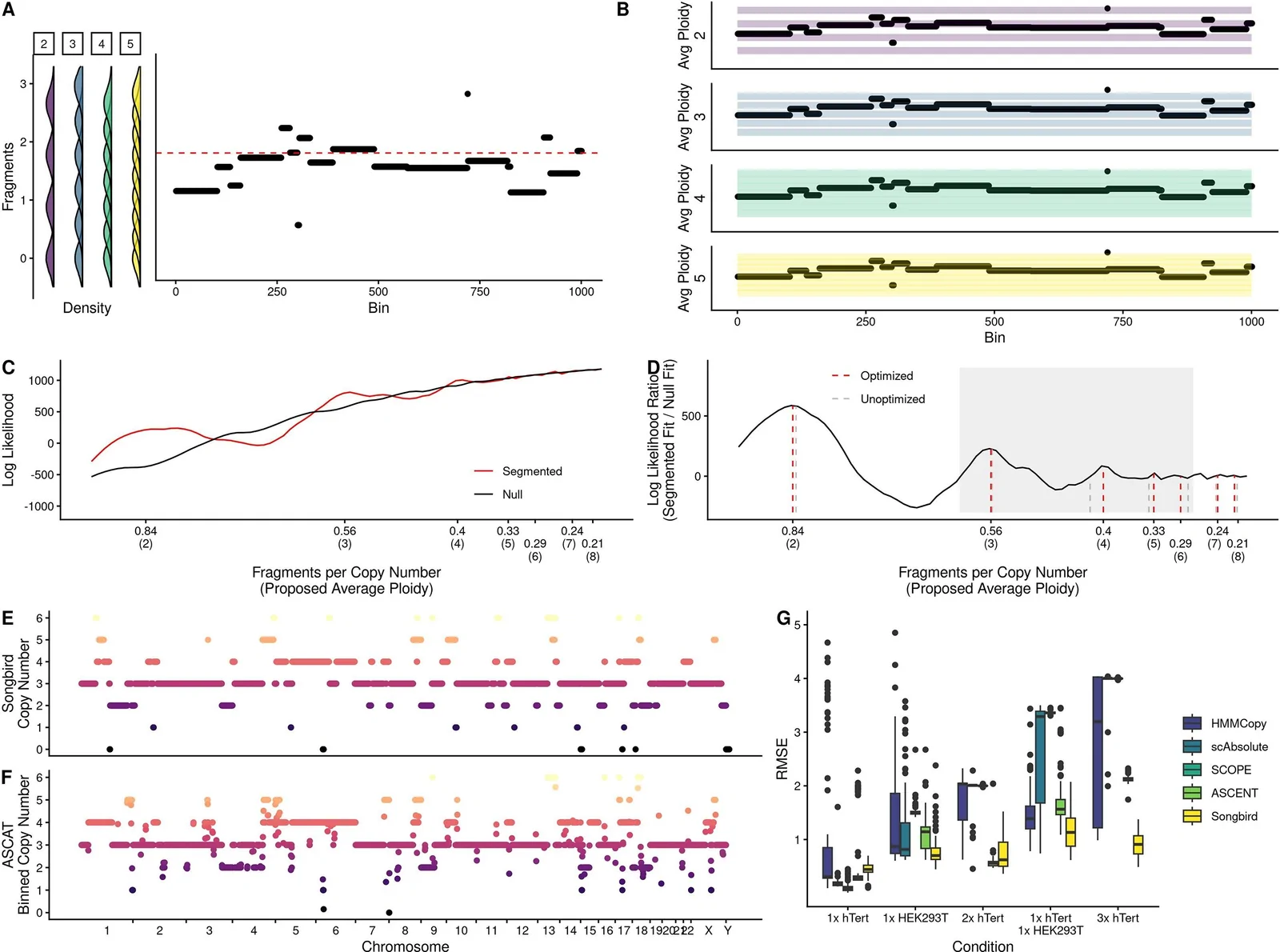
Final copy number optimization. (**A**) Segmented reads from part of an HEK293T Cell. The red line marks the modal segmented read depth across the whole cell, and the distributions on the left depict the probability density if the modal segmented read depth corresponds to copy numbers 2–5. (**B**) Same probability density distributions as in panel (A) except highlighted regions indicate read depths within 1 standard deviation of the implied copy number states. Increasing copy number causes high density regions to overlap. (**C**) Likelihood of fit for the segmented and null data in a HEK293T cell. (**D**) Likelihood ratio of the segmented fit and null fit. Gray dashed lines indicate the initial read depth corresponding to a copy number prior to optimization. Red indicates optimized read depth (**E, F**) Comparison of the final copy number profile for the HEK293T cell compared to the ASCAT ground truth. (**G**) RMSE between the estimated and ground truth copy number profile across the ploidy ladder for various tools.

To validate Songbird’s copy number fitting, outside of the ladder, we used DLP+ sequencing data from a previously published esophageal adenocarcinoma organoid. This organoid displays significant aneuploidy with subclonal populations [39], making a singular ground truth profile difficult to estimate with ASCAT (Fig. 5A). Prior published analysis [39] included karyotyping results, which can serve as qualitative orthogonal evidence supporting aneuploidy analysis; however, it cannot be used as per-bin ground truth to validate the true copy number states (Fig. 5B) [39]. Songbird successfully identifies a varied copy number landscape in the sample (Fig. 5C). Compared to SCOPE, scAbsolute, HMMCopy, and ASCENT, the reads assigned to each copy number by Songbird were more normally distributed, suggesting that reads are assigned to copy number appropriately (Fig. 5D). This is quantified in Fig. 5E by subtracting Shapiro–Wilk test statistic from 1 so that 0 is perfectly normally distributed.

**Figure 5.**
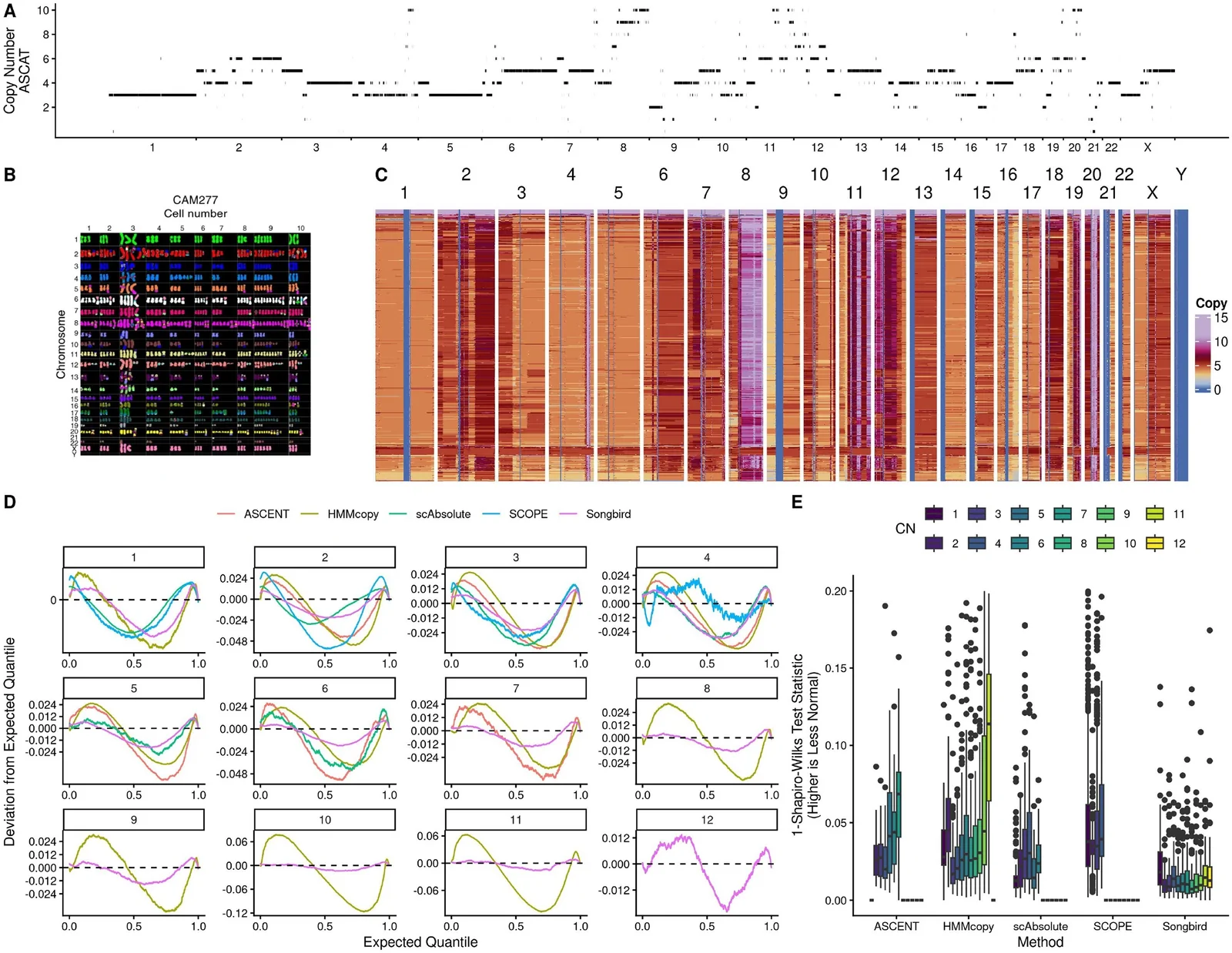
Songbird recovers copy number states of esophageal adenocarcinoma organoids. (**A**) ASCAT copy number for the CAM277 organoid. (**B**) Karyotyping of 10 cells from the organoid showing high and varied copy number states. Figure used with permission from Li *et al*. 2018 [39]. (**C**) Songbird estimated single-cell copy number profile for the CAM277 organoid. (**D**) Normalized quantile–quantile plot for reads assigned to each copy by each tool. The *x-*axis is the expected quantile, and the *y* -axis is the ${\rm observed\, quantile} - {\rm expected\, quantile}$. (**E**) 1-Shapiro–Wilk test for normality for reads assigned to each copy number across all tools compared. Copy numbers that were not called by the various methods were given a zero score.

### Recapitulating single-cell ecDNA dynamics with Songbird

ecDNA and BFBs are two types of SV commonly found in genetically unstable cancers. The copy numbers of these SVs are highly variable from cell to cell, making single-cell profiling important. Due to the mechanisms that form these two SVs, they display distinct copy dynamics. EcDNA are acentromeric so they are inherited stochastically by successor cells. Furthermore, since ecDNA are relatively stable, they generally do not change in composition. As a result, the ratios of fragments in the ecDNA are constant from generation to generation (Fig. 6A). BFBs are formed when a chromosome breaks and is inappropriately fused to a sister chromatid. The resultant inverted chromatid is unstable and likely to break again, which can trigger another fusion event. The breakage–fusion cycle repeats with each breakage, changing the copies of the genetic material in the lesion. Notably, unlike ecDNA, BFBs change in both copy and composition, resulting in a variable ratio of fragments within the lesion (Fig. 6B).

**Figure 6.**
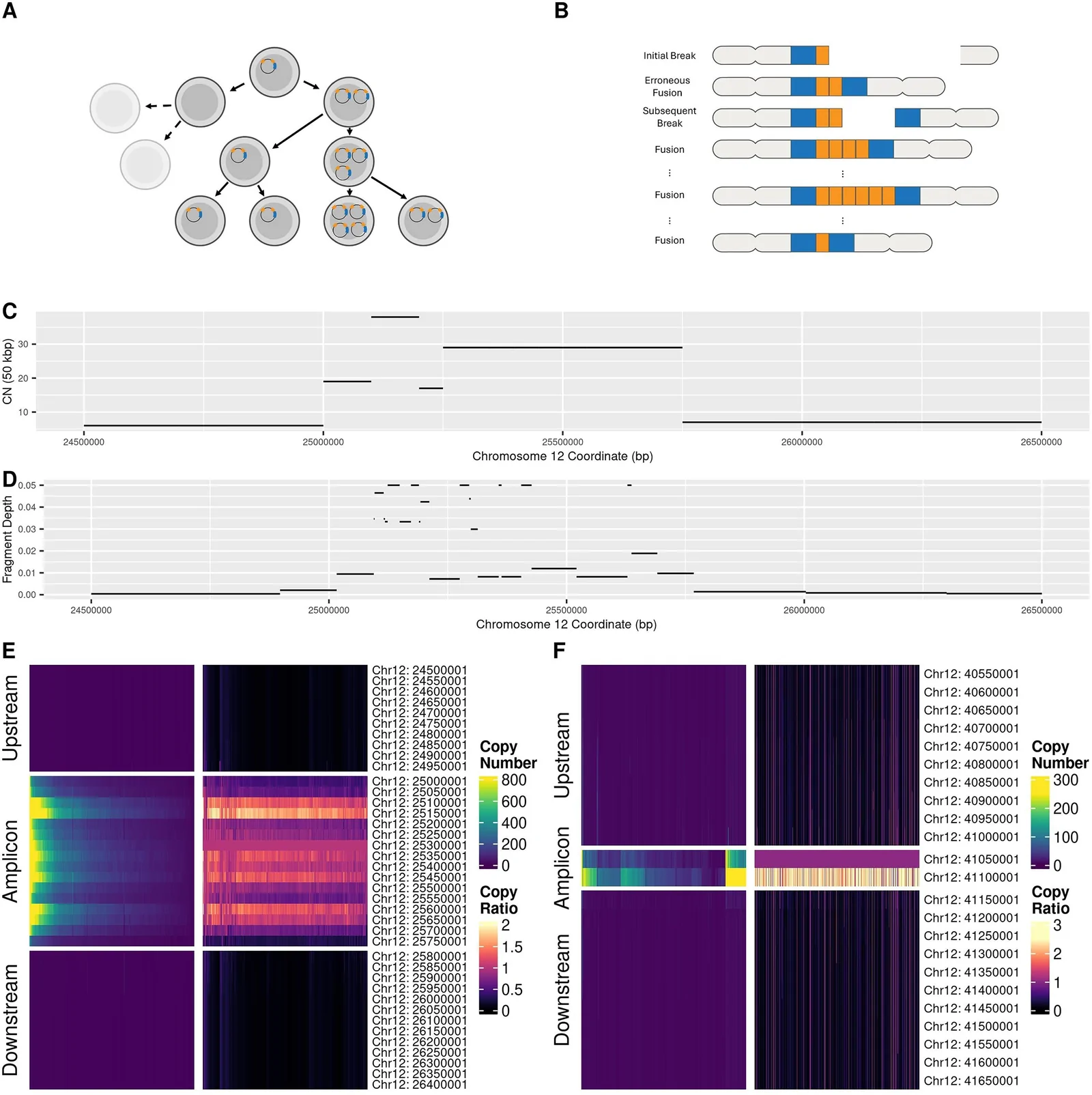
Songbird identifies single-cell dynamics of ecDNA amplifications. (**A**) Cartoon demonstrating inheritance patterns of ecDNA. Notably the fragment ratios stay consistent from generation to generation even as total copy varies. Child cells with 0 ecDNA are omitted for clarity. (**B**) Cartoon demonstrating formation of BFBs. The cycle of breakage followed by fusion in each generation changes the ratio of fragments and the total copy. (**C**) 50 kb copy number profile in one CAM277 cell overlapping a region with a known ecDNA. (**D**) Read depth in the same cell for the true genomic fragments that form the same ecDNA in panels (C) and (D). True breakpoints between the fragments were determined using previously published nanopore long read sequencing [40]. (**E**) 50 kb binned copy number and fragment ratio for the ecDNA across all cells in the CAM277 organoid. (**F**) 50 kb binned copy number and fragment ratio for the BFB across all cells in the CAM277 organoid. The BFB was identified using nanopore long read sequencing in the same manner as the ecDNA [40].

Songbird is able to segment the genome down to 30 kb bins with relatively high fidelity with respect to the 500 kb segmentation (Supplementary Fig. S3D). While the arbitrary bin start and end points do not recapitulate the actual genomic breakpoints of SVs, they can approximate them. Previously, we collected Oxford Nanopore long read sequencing from the same esophageal adenocarcinoma organoid and we used it to reconstruct the structure of an ecDNA and a BFB in that sample [40]. Songbird run at 50 kb bin size approximated the ecDNA breakpoints, identifying the more varied region near the start of the amplicon, and the less varied region further along (Fig. 6C). Identified breakpoints were comparable to the true breakpoints obtained after long read assembly (Fig. 6D). Furthermore, the binned approximation of this ecDNA identified directly from DLP+ data (Fig. 6C) recapitulated the expected inheritance dynamics. The copy number state of ecDNA-containing bins varied between cells, but the ratio between bins remained consistent within each cell (Fig. 6E). In contrast, a BFB further down the chromosome identified by the same long read sequencing does not show consistent ratios between bins, but does display a high and varied copy number between cells. This is consistent with the underlying process driving inheritance of BFBs (Fig. 6F).

## Discussion

Here, we present Songbird, a wavelet-based, WGD-aware copy number caller for scWGS methods, which rely on tagmentation for the initial cleavage and library adapter deposition. We demonstrate that Songbird, using an implementation of the unbalanced Haar wavelet transform, is able to accurately identify copy number breakpoints in simulated DLP+ data, and reliably recover those breakpoints in cells with as little as 0.01× coverage. Previously, Haar-based approaches were used mainly for smoothing or segmentation in array/bulk CNV settings, whereas Songbird adapts unbalanced Haar segmentation to low-coverage Tn5-based scWGS and couples it to whole-genome-doubling/ploidy-aware integer copy number calling. Furthermore, Songbird’s novel ploidy calling algorithm based on the geometry of sequencing fragments is able to recover the true ploidy of cells up to an average ploidy of 6, and consistently estimate the correct ploidy in cells with anywhere from 0.0025× to 0.01× coverage, depending on the cell’s average ploidy (Supplementary Fig. S3E).

Since Songbird’s initial ploidy estimation is performed independently per-cell, and DLP+ has relatively low coverage, Songbird’s initial ploidy estimates tend to be variable, with a slight correlation to overall coverage (Fig. 3E and Supplementary Fig. S3E). Some of the correlation between ploidy estimate and coverage may be attributed to S/G2-phase cells, which have more DNA content and therefore will be overrepresented in the high coverage cells. The high variability in these initial ploidy estimates is reduced by the clustering and ploidy correction process in the final integer fitting as displayed by Songbird’s much more consistent RMSE against the ASCAT copy number calls. Of note, ASCENT displays a more consistent and similarly accurate copy number profile in the 1× and 2× hTERT conditions, as evinced by the lower interquartile range in the RMSE against the ASCAT calls (Fig. 4G).

The hTERT multiples in the ploidy ladder highlighted a key weakness of scWGS copy number callers that only rely on read depth ratio (HMMCopy and SCOPE) as they only estimated an average ploidy of 2 for the 2× and 3× hTERT conditions. This is likely due to a lack of segments with variable copy number states and confirmed by these tools’ better performance in the 1× HEK condition, and HMMCopy’s performance in the 1× hTERT 1× HEK condition. Alleloscope, and scAbsolute both do not rely on read depth ratio for their copy number calls, but both display a strong tendency toward calling copy number two, indicating that the shallow nature of DLP+ data makes phasing or comparing local read density difficult when estimating ploidy. In contrast, Songbird and ASCENT were able to successfully recover the 1× and 2× hTERT conditions. Given that both tools rely on quantifying overlapping fragments, future tools should consider the patterns of fragment overlaps in ploidy estimation.

Beyond the ploidy ladder generated for this paper, we demonstrated Songbird’s versatility to a wide variety of protocols, successfully recapitulating the true ploidy from several other Tn5-based scWGS sequencing methods and in multi-clonal organoid samples. Notably Songbird accurately recapitulates the expected single-cell copy number dynamics of an already known ecDNA.

In developing Songbird, we have identified and partially characterized artifactual read pairs that seem to have interesting properties. These fragments occur far more often than expected by chance, violate the known maximum ploidy in cells, and are largely duplicated in a 1:1 or 1:2 ratio. The first two observations hint at their artifactual nature, and the last seems to indicate that these fragments are generated prior to PCR amplification or within the first cycle. The only steps prior to PCR amplification in the DLP+ protocol are adapter tagmentation, and Tn5 neutralization. As a result, we suspect that these fragments are generated from an incomplete Tn5 library adapter ligation, either from an inactive member in a Tn5 dimer, or a tagmentation event interrupted by neutralization. Either way, this results in one pair of the Tn5 dimer successfully cleaving one strand of an already fragmented piece of genomic DNA, while the other one fails to do so. This successful, failed cleavage pairing would produce two single stranded DNA fragments, a longer one representing the original, complete Tn5 transposase action, and a shorter one from this partially complete Tn5 cleavage. Since the adapter loading on the Tn5 dimer is random, we expect that we only observe half of these events, with the other half depositing same orientation primers and failing to amplify the short fragment.

While we have not performed a biochemical exploration of these events due to their relatively rare occurrence, ASCENT has also noted their presence and removes these fragments in a comparable manner to Songbird [30]. Given their identification and exclusion in two publications, we believe that future improvements on scWGS copy number calling should perform careful filtering of these fragments.

While Songbird seems to perform well, validation has only been done on a few cell lines and one organoid line. Patient data may have much more variable quality and may require further refinement of the tool. Further work can be done to improve the noisy ploidy estimator, such as replacing the overlap counting with a more sophisticated Poisson or binomial sampling model to more accurately capture the true copy number state. Additionally, Songbird currently treats cells as independent events with very little shared information from cell to cell beyond clustering for ploidy estimate correction. A more holistic model that uses information across multiple cells would be useful. This could potentially improve the accuracy of copy number calls at focal amplifications. Currently, at coarse bin sizes, the copy numbers from focal amplification events are estimated with only a single bin of information, which adds noise into the true copy number estimation since those reads are subject to the same sampling error which makes segmentation a necessity.

## Supplementary Material

gkag960_Supplemental_File

## Data Availability

Songbird is available as an R Package at https://Github.com/GastroEsoLab/Songbird and on Zenodo (doi: 10.5281/zenodo.18224777, https://zenodo.org/records/21230403). Code and data used to generate these figures are also available on Zenodo (doi: 10.5281/zenodo.18224049). Fastq files for the hTERT/HEK ladder are available under the NCBI BioProject PRJNA1493277, and the downstream bedpe files required for Songbird are available publicly on Zenodo (doi: 10.5281/zenodo.18224233 ). Sequencing files for the CAM277 esophageal adenocarcinoma organoid are available from the European Genome-Phenome Archive under EGAN00004957712. Files for the scAbsolute Ladder were retrieved from the European Nucleotide Archive project PRJEB61928. Sequencing files for LIANTI and SmoothSeq were retrieved from NCBI BioProject PRJNA379710 and PRJNA633502, respectively.
