# Supplementary material for: Wavelet-based whole genome doubling aware single-cell copy number calling
Source: Nucleic Acids Res. 2026 Oct 9;54(18):gkag960. doi: 10.1093/nar/gkag960 (PMC13650168; doi:10.1093/nar/gkag960)
Supplement: Supplementary file 1 [file gkag960_supplemental_file.pdf]

Wavelet Based Whole Genome Doubling Aware Single Cell Copy Number Calling

Benjamin K. Wesley, Frank Wos, Soren Germer, Jade E.B. Carter, Silas Maniatis, Khanh Dinh, James S. Roche, Timothy R. Chu, Nicolas Robine, Rebecca Fitzgerald, John Lizhe Zhuang, Simon Tavaré, Karol Nowicki-Osuch

July 2026

References

[1] Laks, E., McPherson, A., Zahn, H., Lai, D., et al. (11, 2019) Clonal decomposition and DNA replication states defined by scaled single-cell genome sequencing. *Cell,* **179**, 1207–1221.e22.

[2] Schneider, M. P., Cullen, A. E., Pangonyte, J., Skelton, J., et al. (3, 2024) scAbsolute: measuring single-cell ploidy and replication status. *Genome Biology,* **25**, 62.

[3] Kolbeinsdottir, S., Zachariadis, V., Sommerauer, C., Lohi, O., Heinäniemi, M., and Enge, M. (09, 2025) Absolute copy number aware CNV calling of sub-megabase segments in ultra-low coverage single-cell DNA sequencing data. *Nucleic Acids Research,* **53**(17), gkaf919.

[4] Fan, X., Yang, C., Li, W., Bai, X., et al. (12, 2021) SMOOTH-seq: single-cell genome sequencing of human cells on a third-generation sequencing platform. *Genome Biology,* **22**, 195.

[5] Chen, C., Xing, D., Tan, L., Li, H., et al. (4, 2017) Single-cell whole-genome analyses by Linear Amplification via Transposon Insertion (LIANTI). *Science,* **356**, 189–194.

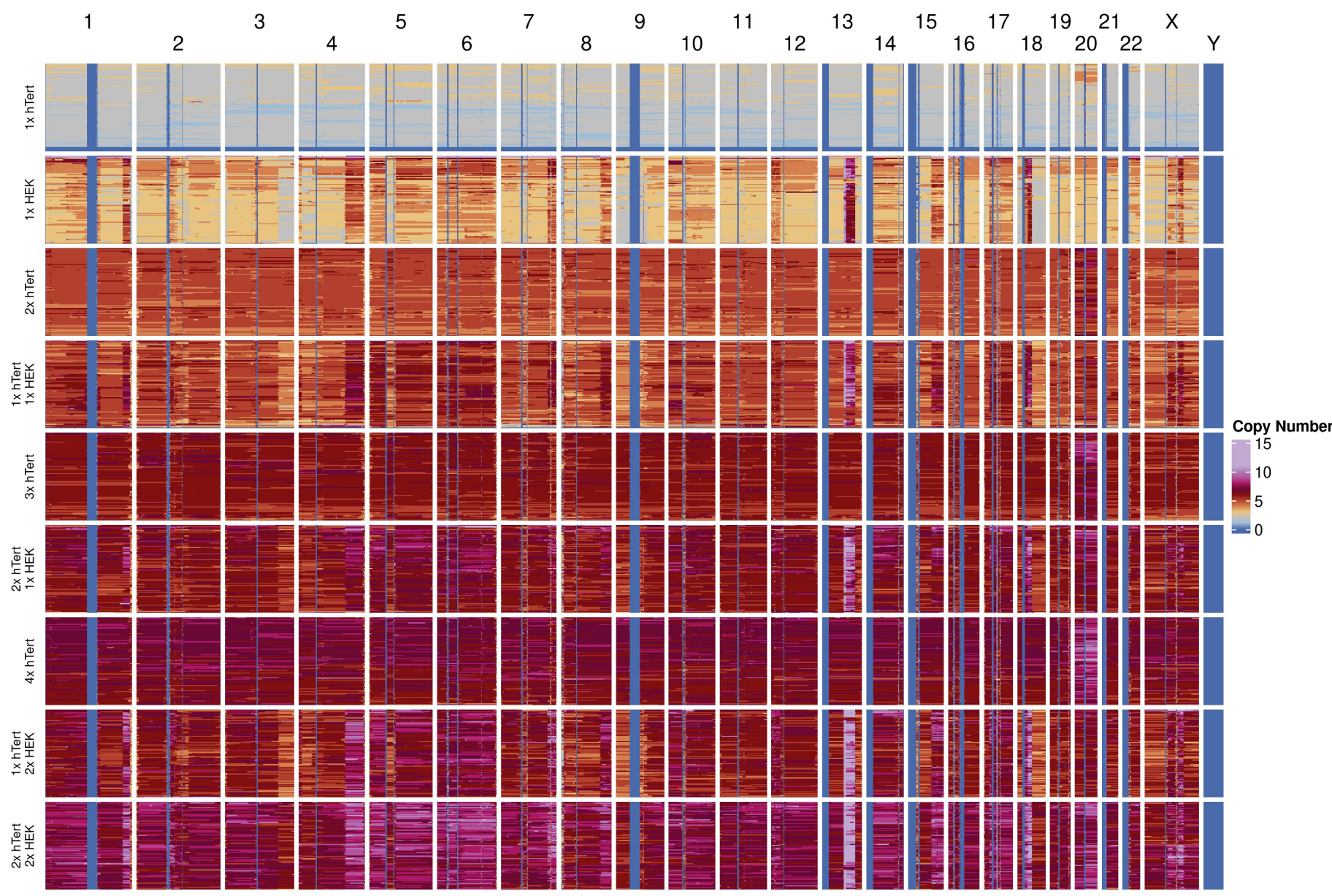

Supplementary Figure 1: Assigned Copy Number profile for the ploidy ladder.

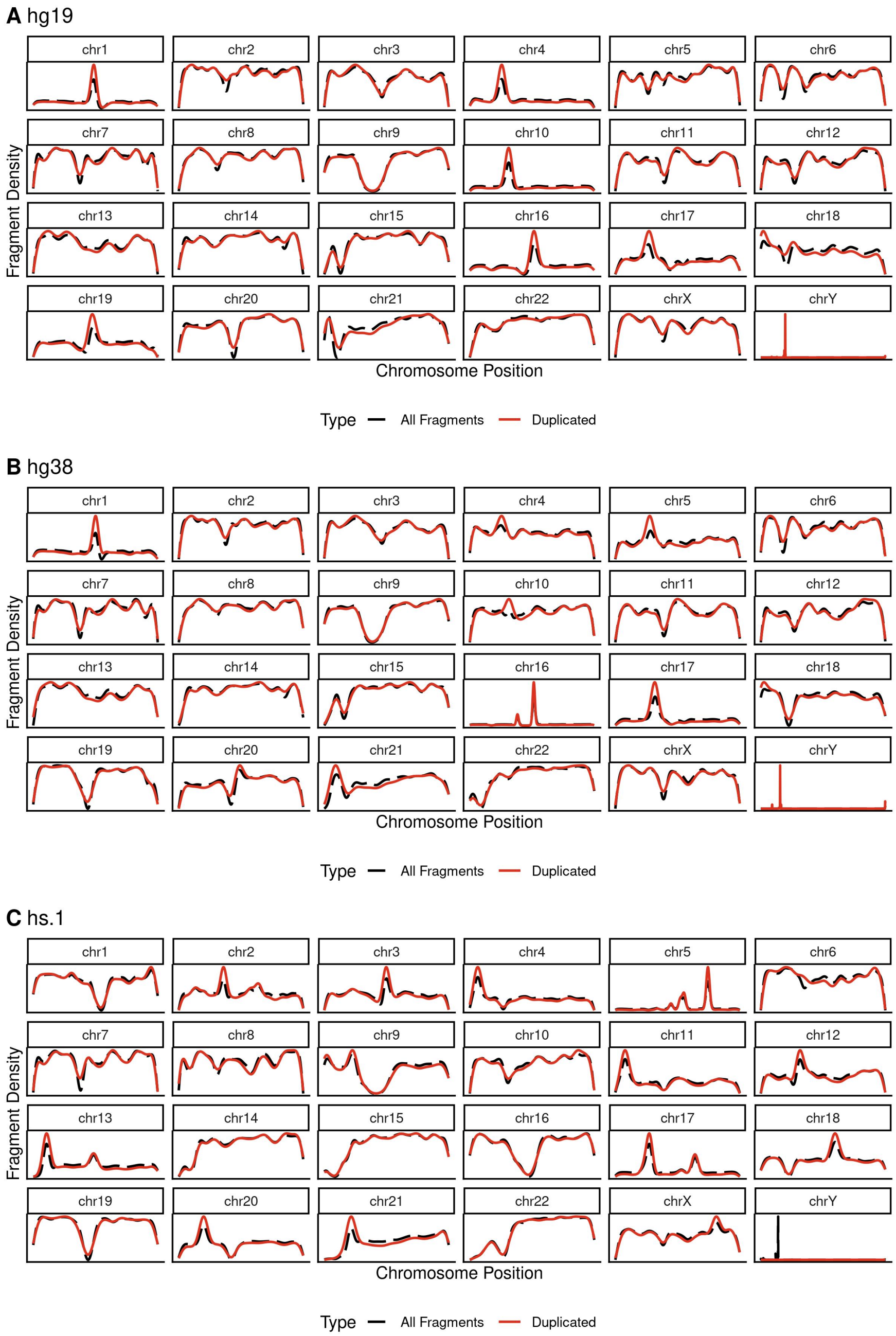

Supplementary Figure 2: Density of artifactual fragments (red) vs all fragments (black dashed) for 100 diploid cells aligned to (A) hg19, (B) hg38, (C) hs.1.

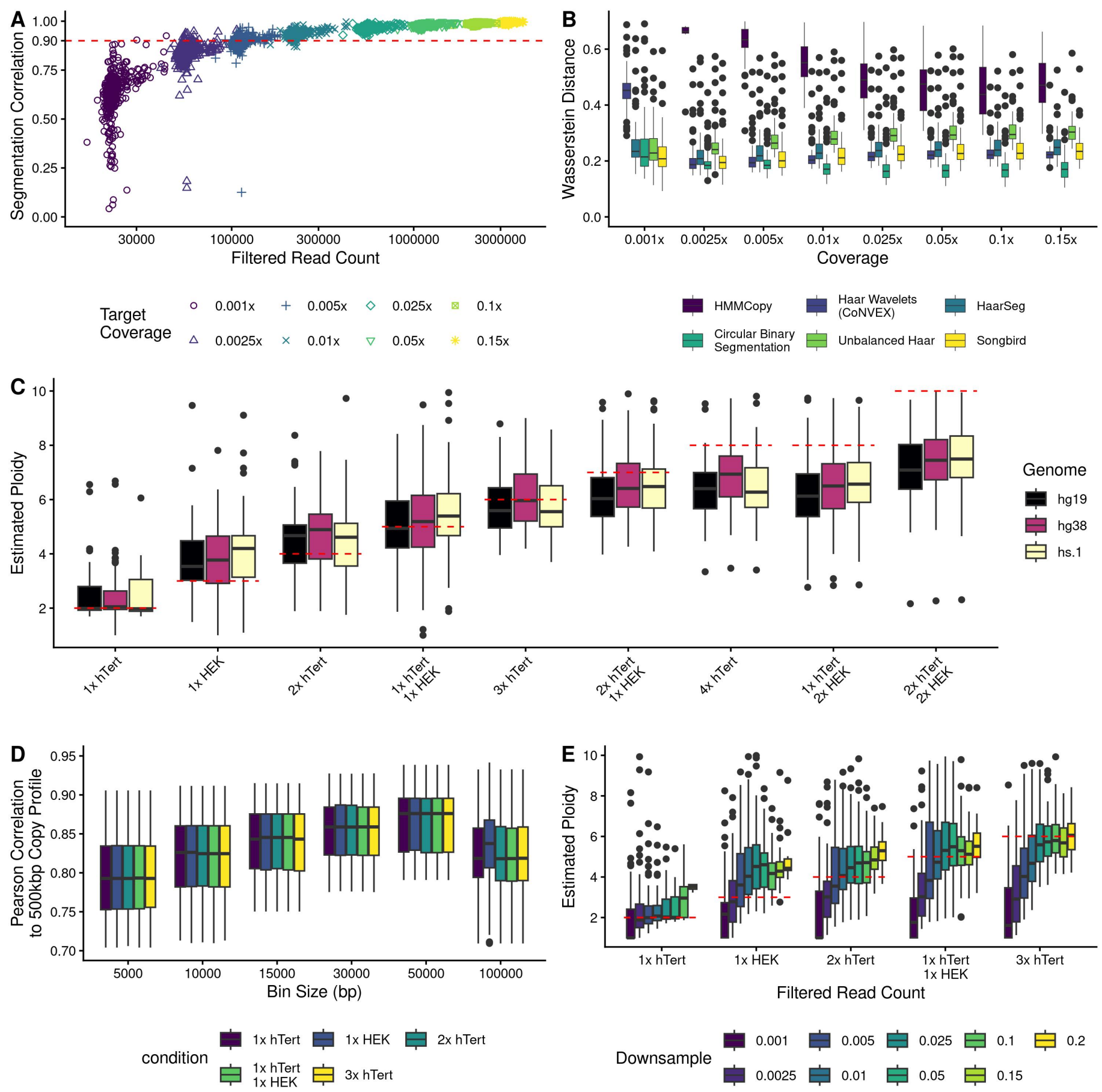

Supplementary Figure 3: (A) Pearson correlation between the original and downsampled segmentation profile for cells in the ploidy ladder. (B) Wasserstein distance between the segmented profile and the true copy number profile for the 1x HEK condition in the ploidy ladder across downsamplings. (C) Estimate Average Ploidy Across the Ploidy Ladder Comparing reads aligned to hg19, hg38, and hs.1. Dashed red line indicates expected ploidy based on the number of cells spotted in each well. (D) Pearson Correlation of the copy number profile compared to 500kbp bin sizes. (E) Estimated Average Ploidy across the ladder across different target depth. Higher target depths exclude cells which do not originally have the required depth.

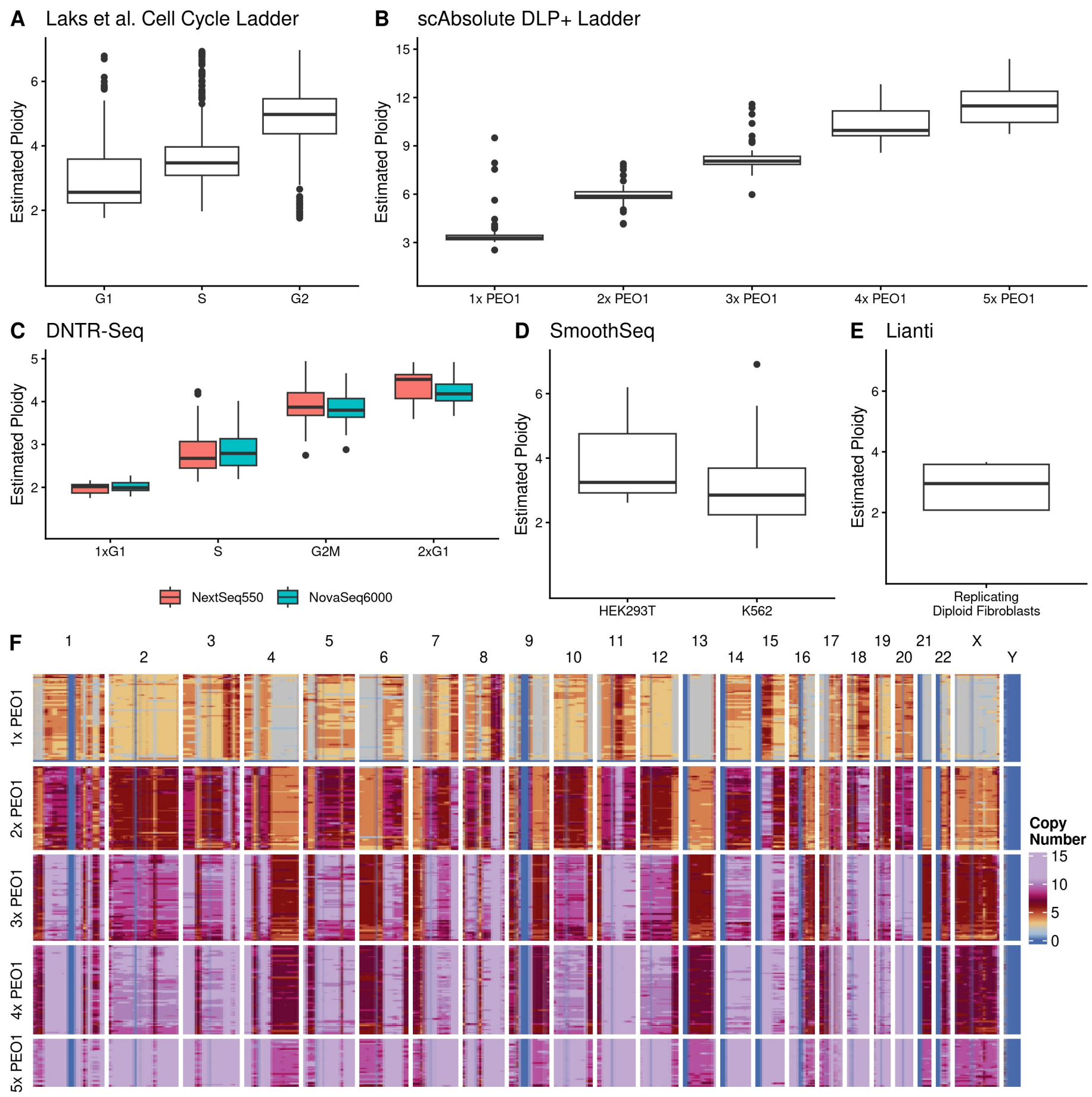

Supplementary Figure 4: (A) Estimated average ploidy for the cell cycle sorted GM18507 cells spotted published in the original DLP+ paper [1]. (B) Estimated average ploidy for the PE01 ladder published as a part of scAbsolute [2]. (C) Estimated average ploidy for cell cycle classified HCT116 cells sequenced using DnTR-Seq [3]. D) Estimated average ploidy for HEK293T and K562 cells sequenced by SmoothSeq [4]. (E) Estimated ploidy for cycling fibroblasts sequenced by LIANTI [5]. (F) Final Copy number profile for the PEO1 cells published alongside scAbsolute [2]
